# Reactive Oxygen and Nitrogen Species in Male Reproductive Health: From Molecular Mechanisms to Clinical Consequences

**DOI:** 10.3390/antiox15070795

**Published:** 2026-06-25

**Authors:** Sijia Wang, Jacqueline Pui Wah Chung, David Yiu Leung Chan

**Affiliations:** 1 Assisted Reproductive Technology Unit, Department of Obstetrics and Gynecology, Faculty of Medicine, The Chinese University of Hong Kong, Hong Kong SAR 999077, China; skye-wangsjj@link.cuhk.edu.hk (S.W.);; 2 Department of Obstetrics and Gynecology, Faculty of Medicine, The Chinese University of Hong Kong, Hong Kong SAR 999077, China

**Keywords:** reactive oxygen species (ROS), reactive nitrogen species (RNS), oxidative stress, male infertility, sperm function, oxidative stress, hormonal regulation, antioxidant therapy

## Abstract

Reactive oxygen species (ROS) and reactive nitrogen species (RNS) are critical modulators of male reproductive health, influencing sperm function, hormonal regulation, and overall fertility. While physiological levels of ROS and RNS are essential for processes such as sperm capacitation and acrosome reaction, their overproduction leads to oxidative and nitrosative stress, contributing to male infertility. Excessive ROS and RNS can damage sperm DNA, proteins, and lipids, impairing motility, viability, and fertilizing capacity. Moreover, these reactive species disrupt the hypothalamic-pituitary-gonadal (HPG) axis, leading to hormonal imbalances that further compromise reproductive function. Environmental factors, lifestyle choices, and underlying health conditions exacerbate the production of ROS and RNS, highlighting the need for preventive and therapeutic strategies. Clinically, ROS- and RNS-mediated redox imbalance has been implicated in several male reproductive disorders, including varicocele, genital tract infection and inflammation, obesity, diabetes and other metabolic disorders, and toxicant-related reproductive dysfunction. Antioxidant supplementation has shown promise in mitigating oxidative stress; however, its efficacy varies, and further research is necessary to establish standardized treatment protocols. These findings underscore the clinical relevance of integrating oxidative stress assessment with conventional semen analysis to improve risk stratification and guide targeted interventions in male infertility. This review synthesizes current knowledge on the molecular mechanisms by which ROS and RNS affect male reproduction and discusses potential clinical interventions to address oxidative and nitrosative stress in male infertility.

## 1. Introduction

Male reproductive health is a fundamental determinant of fertility, with male factors contributing to approximately 50% of infertility cases worldwide [1]. Recent epidemiological data from the Global Burden of Disease study indicate an increasing prevalence of male infertility, a trend most pronounced in populations experiencing rapid urbanization, modern dietary shifts, and heightened exposure to environmental stressors [2,3]. While genetic susceptibility, endocrine disruption, and lifestyle factors (e.g., obesity and smoking) are well-established contributors [4,5,6], clinical translation remains a significant challenge. Currently, standard diagnostic approaches fail to capture the complex molecular etiology of male infertility, thereby hindering the implementation of targeted, mechanism-based therapeutic interventions.

Among the various potential molecular contributors to male infertility, reactive oxygen species (ROS) and reactive nitrogen species (RNS) have emerged as pivotal regulators of both normal and pathological processes within the male reproductive system [7,8,9]. ROS include chemically reactive molecules such as superoxide anion (O_2_•^−^), hydroxyl radical (•OH), and hydrogen peroxide (H_2_O_2_), whereas RNS encompass nitrogen-based species like nitric oxide (NO•) and peroxynitrite (ONOO^−^) [10,11,12]. These reactive species are primarily generated through mitochondrial respiration, NADPH oxidase activity, and inflammatory responses in tissues such as the testes, seminal plasma, and spermatozoa. At physiological levels, ROS and RNS function as essential intracellular signaling mediators that facilitate key steps in fertilization, including sperm maturation, capacitation, hyperactivation, acrosome reaction, and sperm-oocyte fusion [13,14]. Their redox-dependent modulation of tyrosine phosphorylation, membrane fluidity, and calcium signaling is critical for the acquisition of sperm fertilizing competence. However, when their production exceeds the capacity of endogenous antioxidant systems, these species induce oxidative and nitrosative stress, leading to lipid peroxidation, DNA fragmentation, and protein oxidation [15,16]. Such damage compromises sperm motility, viability, and genomic integrity, ultimately impairing male fertility [17,18]. This dual role positions ROS and RNS as double-edged swords—indispensable for normal sperm function, yet detrimental when dysregulated.

Beyond their direct effects on spermatozoa, accumulating evidence suggests that excessive ROS and RNS exert broader influences on the male reproductive microenvironment and endocrine regulation. Oxidative and nitrosative stress can disrupt the structure and function of Sertoli and Leydig cells, impair steroidogenesis, and interfere with paracrine signaling essential for spermatogenic support [19,20]. Moreover, redox imbalance has been implicated in dysregulation of the hypothalamic–pituitary–gonadal (HPG) axis, contributing to altered gonadotropin secretion and testosterone deficiency, thereby linking local testicular damage to systemic hormonal disturbances [21,22].

The production of ROS and RNS within the male reproductive system is influenced by a wide range of endogenous and exogenous factors, including aging, varicocele, infection, metabolic disorders, environmental pollutants, heat exposure, smoking, alcohol consumption, and psychological stress [23]. These diverse insults converge on common redox-sensitive pathways, reinforcing oxidative and nitrosative stress as central mediators of male reproductive dysfunction across heterogeneous clinical conditions. Importantly, such redox disturbances not only affect natural fertility but are also associated with suboptimal outcomes in assisted reproductive technologies, including reduced fertilization rates, impaired embryo development, and increased risk of adverse reproductive outcomes [24,25].

Given the central role of redox imbalance in male infertility, antioxidant-based therapeutic strategies have garnered significant clinical interest. Although antioxidant supplementation has demonstrated potential benefits in reducing oxidative damage and improving selected semen parameters, clinical evidence remains inconsistent, with considerable variability in treatment response [26,27]. These discrepancies likely reflect differences in patient etiology, baseline redox status, antioxidant formulations, dosing regimens, and treatment duration, underscoring the need for a more nuanced understanding of ROS- and RNS-mediated mechanisms in male reproduction. In this review, we integrate current knowledge on the sources, signaling functions, and pathological consequences of ROS and RNS in the male reproductive system, spanning molecular mechanisms to clinical manifestations. By synthesizing experimental and clinical evidence, we aim to clarify the physiological–pathological continuum of redox regulation, critically evaluate existing therapeutic approaches, and highlight emerging directions for precision diagnosis and targeted intervention in male infertility.

## 2. Sources and Regulation of ROS and RNS in the Male Reproductive System

### 2.1. Endogenous Sources

Endogenous production of ROS and RNS is an inherent feature of normal cellular metabolism within the male reproductive system. Under physiological conditions, tightly regulated redox signaling contributes to spermatogenesis, sperm maturation, and fertilization. However, dysregulation of these endogenous sources can lead to excessive ROS and RNS accumulation, overwhelming local antioxidant defenses and resulting in oxidative and nitrosative stress. Multiple cellular compartments contribute to endogenous ROS and RNS generation, with spermatozoa, leukocytes, and somatic cells of the testes and epididymis representing the principal sources.

#### 2.1.1. Sperm Mitochondria

Spermatozoa are a major endogenous source of ROS within the male reproductive tract, primarily through mitochondrial activity [28,29]. During oxidative phosphorylation, electrons are transferred along the mitochondrial electron transport chain to generate ATP required for sperm motility and other energy-dependent processes [30,31]. A small proportion of electrons may prematurely leak from complexes I and III and react with molecular oxygen, forming superoxide anion (O_2_•^−^). This process is amplified when mitochondrial membrane potential is altered, ATP demand is excessive, or mitochondrial integrity is compromised, conditions frequently observed in dysfunctional or immature sperm [32,33,34,35].

In addition to mitochondrial electron leakage, spermatozoa possess enzyme systems capable of generating ROS in a regulated manner. NADPH oxidase (NOX) isoforms expressed in sperm membranes contribute to controlled superoxide and hydrogen peroxide production, which plays a role in capacitation-associated signaling cascades [36,37,38]. Similarly, xanthine oxidase activity, although less prominent than mitochondrial sources, can contribute to ROS generation under pathological conditions, particularly when purine metabolism is dysregulated [39]. Xanthine oxidase catalyzes the oxidation of hypoxanthine and xanthine to uric acid, a process that generates superoxide and hydrogen peroxide as by-products [40]. Enhanced xanthine oxidase activity has been reported in conditions associated with cellular stress, inflammation, or ischemia–reperfusion, thereby amplifying local oxidative burden within the male reproductive tract [41,42]. While these enzymatic sources participate in physiological redox signaling, their overactivation or dysregulation results in excessive ROS production, promoting lipid peroxidation of the sperm plasma membrane and impairing motility and fertilizing capacity.

Nitric oxide (NO•) is generated in spermatozoa and the male reproductive tract through nitric oxide synthase (NOS) activity, with both constitutive isoforms (nNOS and eNOS) and inducible isoform (iNOS) detected in sperm and associated reproductive tissues [43,44]. Nitric oxide (NO•) exerts concentration- and time-dependent effects on sperm function. At physiological levels (generally <1 μM), NO enhances sperm motility and fertilization-related processes by promoting capacitation and acrosome reaction through activation of soluble guanylate cyclase (sGC), cGMP-dependent signaling pathways, and modulation of protein tyrosine phosphorylation [45,46,47,48,49]. In contrast, supraphysiological NO levels (≥1 μM), particularly under conditions of sustained exposure or concomitant superoxide generation, promote nitrosative stress through the formation of peroxynitrite (ONOO^−^), a highly reactive nitrogen species. Peroxynitrite induces protein tyrosine nitration, mitochondrial dysfunction, lipid peroxidation, and DNA damage, ultimately impairing sperm function and viability [50,51,52]. Elevated peroxynitrite formation has been correlated with increased protein tyrosine nitration and decreased sperm motility in pathological conditions where oxidative and nitrosative stress are elevated. Consistently, impaired PRDX6 peroxidase activity has been shown to increase protein tyrosine nitration in spermatozoa, supporting the role of antioxidant peroxidase systems in limiting nitrosative damage [53].

#### 2.1.2. Leukocytes in Seminal Plasma

Leukocytes represent another major endogenous source of and RNS in the male reproductive system, particularly within seminal plasma. Under physiological conditions, baseline leukocyte populations—primarily macrophages and neutrophils—are maintained at low concentrations (typically <1 × 10^6^ cells/mL in human ejaculate) to facilitate immune surveillance and tissue homeostasis. Within the testicular microenvironment, these cells are frequently characterized using techniques such as flow cytometry and immunohistochemistry [54]. Specifically, testicular macrophages, often identified by CD68 expression, play a critical role in maintaining the immune-privileged status of the testes. Through paracrine signaling, they support Leydig cell steroidogenesis, thereby contributing to the regulation of normal spermatogenesis. In this healthy, homeostatic state, leukocyte-derived ROS production remains low and strictly regulated [55].

However, activated leukocytes generate substantially higher amounts of ROS and RNS than spermatozoa [56,57]. Through respiratory burst activity mediated by NADPH oxidase complexes and inducible nitric oxide synthase (iNOS), leukocytes produce large quantities of superoxide, hydrogen peroxide, and nitric oxide. Inflammatory conditions of the male reproductive tract—such as genital tract infections, prostatitis, or epididymitis—promote leukocyte recruitment and activation, resulting in disproportionate ROS and RNS release into seminal plasma [58,59]. In parallel, activated leukocytes secrete a spectrum of pro-inflammatory cytokines, including interleukin-1α (IL-1α), IL-6, IL-8, tumor necrosis factor-α (TNF-α), and colony-stimulating factors, which can further amplify oxidative stress by stimulating ROS-producing pathways and sustaining local inflammation [60,61].

Clinical studies have demonstrated that leukocytospermia is associated with elevated levels of specific cytokines—most notably IL-8, IL-1α, and TNF-α—which are strongly correlated with markers of leukocyte activation such as granulocyte elastase [62,63]. Importantly, increased cytokine concentrations and leukocyte-derived inflammatory mediators may be detected even in men with otherwise normal semen parameters, indicating that inflammatory redox imbalance can occur independently of overt oligozoospermia or asthenozoospermia. This observation underscores that oxidative stress driven by immune activation may represent a functional defect not captured by conventional semen analysis [7,61,63,64].

Excess leukocyte-derived ROS and RNS readily diffuse within seminal plasma and exert deleterious effects on nearby spermatozoa, including lipid peroxidation of the sperm plasma membrane, oxidative modification of structural and enzymatic proteins, and induction of nuclear and mitochondrial DNA damage [15,55,65]. Concurrently, the male reproductive tract is equipped with a complex network of immunoregulatory mechanisms that restrain leukocyte activation and preserve immune tolerance toward germ cells. In addition to non-classical human leukocyte antigen (HLA) class Ib molecules such as HLA-G, which exert inhibitory effects on natural killer cells and cytotoxic T lymphocytes, several soluble and cellular mediators contribute to local immune homeostasis [66]. Anti-inflammatory cytokines, including interleukin-10 (IL-10) and transforming growth factor-β (TGF-β), are present in seminal plasma and act to suppress excessive pro-inflammatory cytokine production and leukocyte activation [67]. Prostaglandins, particularly prostaglandin E_2_ (PGE_2_), further modulate immune responses by inhibiting neutrophil function and shifting cytokine balance toward an anti-inflammatory profile [68,69].

Additional regulatory pathways involve immune checkpoint molecules and apoptosis-inducing signals, such as Fas ligand (FasL) expressed by Sertoli cells, which promotes deletion of activated immune cells and contributes to testicular immune privilege [70,71]. Indoleamine 2,3-dioxygenase (IDO), expressed in testicular and epididymal tissues, may further limit local immune activation by depleting tryptophan and suppressing T-cell proliferation [72,73]. Collectively, these immunomodulatory mechanisms act in concert to limit inflammation-induced oxidative stress and protect spermatozoa from immune-mediated damage. Disruption of this finely balanced regulatory network—through infection, inflammation, or tissue pathology—can shift the seminal microenvironment toward a pro-inflammatory, pro-oxidant state, facilitating sustained leukocyte-derived ROS and RNS production and exacerbating sperm oxidative injury.

Collectively, these findings indicate that leukocyte-driven oxidative and nitrosative stress reflects not only increased ROS/RNS production but also a complex interplay between inflammatory cytokines, immune regulation, and redox signaling. Importantly, elevated oxidative stress markers and sperm DNA fragmentation have also been reported in infertile men with normozoospermic semen profiles, indicating that conventional semen analysis may fail to capture functional redox-related sperm defects [74].

#### 2.1.3. Testicular and Epididymal Cells

Somatic cells within the testes and epididymis constitute additional endogenous sources of ROS and RNS, contributing to both physiological redox signaling and pathological oxidative stress [9,75]. In the testes, Sertoli cells, Leydig cells, and peritubular myoid cells generate ROS as unavoidable by-products of cellular metabolism, steroidogenesis, and active paracrine signaling [76]. Sertoli cells, which provide metabolic and structural support to developing germ cells, produce ROS through mitochondrial respiration and redox-dependent signaling pathways involved in germ cell differentiation and blood–testis barrier dynamics [77,78,79]. Tight regulation of Sertoli cell redox status is therefore essential to maintain a supportive microenvironment for spermatogenesis.

Leydig cells are particularly sensitive to redox imbalance, as mitochondrial and microsomal processes involved in testosterone biosynthesis are intrinsically linked to ROS generation. Enzymatic reactions catalyzed by cytochrome P450 enzymes during steroid hormone synthesis generate superoxide and hydrogen peroxide, necessitating robust antioxidant systems to preserve mitochondrial integrity and steroidogenic capacity [80,81]. Disruption of redox homeostasis in Leydig cells—through excessive ROS production or impaired antioxidant defenses—can reduce testosterone synthesis, alter paracrine signaling within the testis, and indirectly compromise spermatogenic support [82,83,84]. Peritubular myoid cells also contribute to the testicular redox milieu through metabolic activity and regulation of seminiferous tubule contractility [85,86]. Although their role in ROS and RNS production is less pronounced, oxidative stress in these cells can affect extracellular matrix composition and tubule dynamics, potentially influencing germ cell transport and testicular architecture.

Within the epididymis, epithelial cells actively generate ROS and RNS at tightly controlled levels, which are essential for sperm maturation, membrane remodeling, and acquisition of progressive motility [87,88]. Epididymal epithelial cells secrete antioxidants and redox-active molecules that fine-tune the luminal environment, supporting disulfide bond formation in sperm surface proteins and stabilization of sperm chromatin [89,90,91]. Because spermatozoa are transcriptionally and translationally inactive during epididymal transit, they rely heavily on the epididymal microenvironment to regulate redox balance and complete functional maturation [92,93].

Recent evidence from a cadmium-exposed prepubertal rat model further supports the sensitivity of testicular and epididymal tissues to toxicant-induced oxidative stress. Cadmium exposure was shown to accumulate in the testes and epididymides, reduce serum testosterone, impair antioxidant enzyme activity, and disrupt testicular and epididymal epithelial organization [94]. Conversely, epigallocatechin-3-gallate partially restored antioxidant activity, reduced cadmium accumulation, and improved testicular and epididymal histology, suggesting that antioxidant protection is important for preserving the structural and functional integrity of these reproductive tissues [94]. These findings reinforce that testicular and epididymal cells are not only regulators of physiological redox signaling but also vulnerable targets of environmental toxicants.

Dysregulation of ROS and RNS production in the epididymis—arising from aging, inflammation, infection, or exposure to environmental toxicants—can disrupt this finely tuned redox control [37,87]. Excessive oxidative or nitrosative stress in the epididymal lumen may impair sperm membrane integrity, promote oxidative DNA damage, and alter chromatin packaging before ejaculation, thereby predisposing spermatozoa to reduced fertilizing capacity and compromised reproductive outcomes [95,96]. Collectively, these endogenous sources highlight the complexity of redox regulation within the male reproductive system. While physiological ROS and RNS generation is essential for normal reproductive function, disruption of the delicate balance between production and antioxidant defense leads to oxidative and nitrosative stress, forming a mechanistic bridge between cellular dysfunction and clinical manifestations of male infertility [97].

#### 2.1.4. Stage-Specific Generation of ROS and RNS During Spermatogenesis

Spermatogenesis is a highly coordinated process involving mitotic proliferation of spermatogonia, meiotic division of spermatocytes, spermiogenesis, spermiation, and subsequent epididymal maturation. ROS and RNS are generated in a stage-dependent manner throughout these processes, reflecting changes in mitochondrial activity, chromatin remodeling, membrane restructuring, and somatic cell–germ cell interactions [75]. During the spermatogonial and early spermatocyte stages, low levels of ROS are mainly produced through mitochondrial respiration and redox-sensitive enzymatic systems in germ cells and adjacent Sertoli cells [37]. At physiological levels, these reactive species participate in signaling pathways that regulate spermatogonial proliferation, differentiation, and meiotic entry; however, excessive ROS may impair germ cell survival, disrupt Sertoli cell support, and promote apoptosis of developing germ cells.

During meiosis and spermiogenesis, increased metabolic activity, chromosomal recombination, and extensive structural remodeling render germ cells particularly vulnerable to redox imbalance. ROS generated by mitochondrial electron leakage and testicular somatic cells may induce oxidative DNA lesions when antioxidant defenses are insufficient. In parallel, nitric oxide produced by NOS isoforms in germ cells and somatic cells may modulate meiotic progression and redox-sensitive signaling [9]. Under inflammatory or pathological conditions, excessive NO can react with superoxide to form peroxynitrite, leading to nitrosative stress, protein tyrosine nitration, and impaired germ cell development. During spermiogenesis, elongating spermatids undergo acrosome formation, flagellar development, mitochondrial sheath organization, cytoplasmic elimination, and chromatin condensation [98]. Because developing spermatids progressively lose cytoplasm and intrinsic antioxidant capacity, excessive ROS/RNS during this period may disrupt protamine replacement, mitochondrial organization, and flagellar formation, resulting in sperm with abnormal morphology, reduced motility, or increased DNA fragmentation.

After spermiation, spermatozoa acquire functional competence during epididymal maturation, where controlled redox signaling supports membrane remodeling, disulfide bond formation, chromatin stabilization, and progressive motility [88]. Epididymal epithelial cells contribute to the regulation of ROS/RNS within the luminal environment while supplying antioxidant protection to maturing spermatozoa [88]. Disruption of this balance by aging, inflammation, infection, varicocele, metabolic stress, or toxicant exposure may shift physiological redox signaling toward oxidative and nitrosative injury before ejaculation. Thus, ROS and RNS generation during spermatogenesis should be viewed as a stage-specific and tightly regulated process: physiological redox signaling supports germ cell development and sperm maturation, whereas excessive or sustained ROS/RNS production contributes to defective spermatogenesis and impaired sperm function.

### 2.2. Exogenous and Environmental Sources of ROS and RNS

In addition to endogenous production, a wide range of exogenous and environmental factors contribute to excessive generation of ROS and RNS in the male reproductive system. These external influences often act by amplifying intrinsic redox pathways, impairing antioxidant defenses, or inducing chronic inflammation, thereby exacerbating oxidative and nitrosative stress. Lifestyle habits, environmental exposures, and systemic pathological conditions represent major categories of exogenous contributors implicated in male reproductive dysfunction.

#### 2.2.1. Lifestyle Factors

Lifestyle-related factors are among the most prevalent and modifiable sources of oxidative and nitrosative stress in men, exerting their effects through systemic metabolic alterations, local inflammation, and direct redox insults to the male reproductive tract. Cigarette smoking is a well-established contributor, as tobacco smoke contains a high burden of free radicals, reactive aldehydes, and transition metals that directly increase systemic and seminal ROS levels [99,100]. In addition to exogenous oxidants, smoking induces endogenous ROS production by activating inflammatory pathways and impairing mitochondrial function in reproductive tissues [101,102]. Clinically, smoking has been consistently associated with elevated seminal oxidative stress, increased sperm DNA fragmentation, reduced motility, and abnormal morphology [103,104]. Furthermore, smoking-related inflammation promotes leukocyte recruitment and activation within seminal plasma, leading to excessive production of superoxide and nitric oxide via NADPH oxidase and inducible nitric oxide synthase pathways, thereby amplifying oxidative and nitrosative stress beyond the intrinsic capacity of seminal antioxidant defenses [105,106].

Alcohol consumption represents another important lifestyle-related source of redox imbalance. Chronic or excessive alcohol intake disrupts mitochondrial bioenergetics and redox homeostasis through ethanol metabolism, which generates acetaldehyde and increases the NADH/NAD^+^ ratio, favoring electron leakage from the mitochondrial respiratory chain and enhanced ROS formation [107,108]. Alcohol-induced oxidative stress also promotes lipid peroxidation, alters membrane composition, and impairs antioxidant enzyme activity [109,110,111]. Within the testes, these effects can suppress Leydig cell steroidogenesis, reduce testosterone production, and compromise Sertoli cell support of spermatogenesis [104,112,113]. Experimental and clinical studies indicate that prolonged alcohol exposure negatively affects sperm concentration, motility, and DNA integrity [112,114,115]. Even moderate alcohol consumption may exert cumulative oxidative effects in susceptible individuals, particularly when combined with other risk factors such as smoking, obesity, or micronutrient deficiency [104,116,117].

Obesity and poor dietary habits are increasingly recognized as key drivers of oxidative and inflammatory stress in male infertility. Excess adiposity is characterized by chronic low-grade inflammation, increased secretion of pro-inflammatory adipokines, and elevated systemic oxidative stress [118]. Adipose tissue–derived cytokines and free fatty acids promote mitochondrial dysfunction and ROS overproduction in peripheral tissues, including the testes [119,120]. Obesity-associated insulin resistance and hormonal dysregulation—such as reduced testosterone and increased estrogen levels—further exacerbate redox imbalance and impair spermatogenic efficiency [121]. In parallel, diets deficient in essential micronutrients and dietary antioxidants limit the capacity of endogenous antioxidant systems, including enzymatic and non-enzymatic defenses in seminal plasma [122,123]. This deficiency renders spermatozoa particularly vulnerable to oxidative and nitrosative damage, given their high content of polyunsaturated fatty acids and limited intrinsic antioxidant capacity [124]. Collectively, these lifestyle-related factors converge on shared redox-sensitive pathways, linking metabolic health, inflammation, and oxidative stress to impaired male reproductive function.

#### 2.2.2. Environmental Exposures

Environmental pollutants constitute a major source of exogenous oxidative and nitrosative stress affecting male reproductive health, acting through systemic inflammatory responses, direct redox imbalance, and disruption of endocrine and mitochondrial function [125,126]. Air pollution, particularly fine particulate matter (PM_2_._5_ and PM_10_), polycyclic aromatic hydrocarbons (PAHs), and traffic-related nitrogen oxides, has been consistently linked to increased systemic oxidative stress and impaired semen quality [127,128]. Upon entry into the body, many airborne pollutants undergo metabolic activation and redox cycling, generating quinones and semiquinone radicals that act as potent oxidizing agents, thereby amplifying intracellular ROS production. Excessive ROS generated through these pathways induces lipid peroxidation of cellular membranes and enhances oxidative DNA damage, including strand breaks and base modifications [129]. In parallel, oxidative stress facilitates the binding and bioactivation of PAHs via aryl hydrocarbon receptor–dependent pathways and promotes the formation of PAH–DNA adducts, further compromising genomic integrity. These pollutants can penetrate pulmonary and vascular barriers, enter systemic circulation, and accumulate in reproductive tissues, where they activate inflammatory signaling and stimulate ROS-generating enzymes such as NADPH oxidases. Epidemiological and experimental studies have demonstrated that exposure to ambient air pollution is associated with reduced sperm concentration and motility, increased sperm DNA fragmentation, and altered chromatin packaging, supporting a mechanistic link between environmental air quality, oxidative stress, and male reproductive dysfunction [130,131,132].

Exposure to heavy metals such as lead, cadmium, mercury, and arsenic is strongly associated with oxidative and nitrosative stress in the male reproductive system [133,134,135]. These metals exert toxicity through multiple redox-related mechanisms, including disruption of mitochondrial electron transport, displacement of essential trace elements, inhibition of antioxidant enzymes (such as superoxide dismutase and glutathione peroxidase), and promotion of free radical generation via Fenton-like reactions [136,137,138]. In addition, heavy metals can deplete glutathione reserves and bind to protein sulfhydryl groups, thereby weakening thiol-dependent antioxidant defenses and increasing susceptibility to lipid peroxidation, protein oxidation, and DNA damage. Cadmium and lead may also impair Sertoli cell junctional integrity and Leydig cell steroidogenic function, linking metal-induced oxidative stress to blood–testis barrier disruption, reduced testosterone synthesis, and defective spermatogenic support. Accumulation of heavy metals in the testes and epididymis interferes with spermatogenesis, impairs steroidogenesis, induces sperm DNA strand breaks, and promotes lipid peroxidation of sperm membranes. In addition to direct genotoxic effects, heavy metals have been shown to alter DNA methylation patterns and histone modifications in germ cells, suggesting a role in epigenetic dysregulation and raising concern for potential transgenerational reproductive effects [139].

Radiation exposure, including ionizing radiation from medical imaging, radiotherapy, and occupational sources, as well as prolonged exposure to non-ionizing electromagnetic radiation, induces ROS generation through radiolysis of intracellular water and secondary mitochondrial injury [140,141]. The resulting hydroxyl radicals, superoxide, and hydrogen peroxide cause oxidative DNA damage, base modifications, and chromosomal instability in germ cells. These effects are particularly detrimental in the testes due to the high proliferative rate of spermatogonia and the limited capacity of mature spermatozoa to repair DNA lesions. Even subclinical or fractionated radiation exposure has been associated with long-term alterations in sperm DNA integrity and reduced fertility potential [142].

Endocrine-disrupting chemicals (EDCs), such as phthalates, bisphenols (e.g., BPA), and certain pesticides, represent another important class of environmental agents capable of inducing oxidative and nitrosative stress [143,144]. Beyond their direct interference with androgen and estrogen signaling, EDCs stimulate ROS and RNS production through mitochondrial dysfunction, disruption of calcium homeostasis, and activation of pro-inflammatory pathways [145,146]. EDC-induced oxidative stress has been implicated in impaired Sertoli and Leydig cell function, abnormal spermatogenesis, and compromised sperm DNA integrity [147]. Given their widespread presence in consumer products, food packaging, and agricultural residues, chronic low-level exposure to EDCs is increasingly recognized as a contributor to cumulative reproductive toxicity and declining male fertility at the population level.

#### 2.2.3. Infections and Systemic Diseases

Infectious and systemic disease states are important drivers of exogenous ROS and RNS generation in male infertility, largely by creating a sustained inflammatory microenvironment in the genital tract and/or systemic metabolic stress that perturbs testicular function [148]. Male reproductive tract infections (e.g., prostatitis, epididymitis, accessory gland inflammation) provoke leukocyte recruitment and activation, and activated neutrophils/macrophages generate high levels of ROS through the respiratory burst (NADPH oxidase–dependent superoxide production with downstream hydrogen peroxide formation) and RNS via inducible nitric oxide synthase (iNOS)–derived nitric oxide. The interaction of NO• with superoxide promotes peroxynitrite (ONOO^−^) formation, linking inflammatory activation to nitrosative stress and protein nitration [149,150]. In addition, leukocyte-derived oxidants can propagate secondary damage through myeloperoxidase-dependent reactions and lipid peroxidation cascades, amplifying sperm membrane injury and DNA oxidation. Importantly, oxidative stress may be elevated even in “borderline” or intermittent leukocytospermia or in clinically subtle inflammatory states, supporting the concept that inflammatory oxidative injury can occur without overt abnormalities on routine semen analysis.

Systemic metabolic disorders, particularly diabetes mellitus, are strongly linked to oxidative and nitrosative stress–mediated reproductive dysfunction. Under hyperglycemic conditions, excessive mitochondrial ROS production in testicular and vascular cells overwhelms endogenous antioxidant defenses, shifting the redox balance toward oxidative stress [151,152]. While low physiological levels of ROS are required for normal sperm maturation, capacitation, and fertilization, diabetes-associated ROS excess induces lipid peroxidation of the sperm plasma membrane, compromises membrane fluidity, and disrupts mitochondrial function [153]. Oxidative stress also damages nuclear and mitochondrial DNA, promoting strand breaks, base oxidation, and activation of apoptotic pathways in germ cells. In parallel, redox-sensitive signaling pathways, including PI3K/AKT/mTOR, may be dysregulated under hyperglycemic stress, leading to abnormal autophagy, impaired blood–testis barrier integrity, and reduced Leydig and Sertoli cell support of spermatogenesis [154,155,156]. Clinically and experimentally, these processes are consistently associated with reduced sperm motility, increased DNA fragmentation, and altered chromatin organization, reflecting the heightened vulnerability of mature spermatozoa to oxidative damage and their limited capacity for DNA repair [157,158].

Varicocele represents a paradigmatic condition linking testicular hemodynamic disturbance to oxidative stress–mediated male infertility. Impaired venous drainage and reflux lead to testicular hyperthermia, altered oxygenation, and metabolic stress, which collectively promote mitochondrial dysfunction and excessive ROS generation in the testes and epididymis [159,160]. Clinically, varicocele is consistently associated with elevated ROS levels and reduced total antioxidant capacity (TAC) in seminal plasma, with oxidative stress severity correlating with varicocele grade and being further exacerbated by concomitant infection or inflammation. Increased levels of oxidative stress markers, including malondialdehyde and nitric oxide, alongside reduced nonenzymatic and enzymatic antioxidant defenses, reflect a disrupted redox balance [161,162]. This imbalance contributes to sperm morphological abnormalities, residual cytoplasm retention, lipid peroxidation, and oxidative DNA damage. Importantly, surgical varicocelectomy has been shown to partially restore redox homeostasis by reducing ROS burden and normalizing multiple oxidative stress biomarkers, concomitant with improvements in sperm quality [163,164]. Together, these findings support oxidative stress as a central mechanistic mediator linking vascular pathology to impaired spermatogenesis and reduced fertility potential in men with varicocele.

### 2.3. Antioxidant Defense Mechanisms

To counterbalance ROS and RNS production, the male reproductive system relies on a multi-layered antioxidant network distributed across seminal plasma, epididymal/testicular epithelia, and spermatozoa. This organization is particularly important because mature sperm have minimal cytoplasm and therefore limited capacity to synthesize or regenerate antioxidant defenses de novo, making them highly dependent on extrinsic antioxidants and redox buffering in the luminal environment [165,166]. Antioxidants act by (i) preventing the accumulation of primary ROS (e.g., superoxide), (ii) detoxifying secondary oxidants (e.g., hydrogen peroxide and lipid hydroperoxides), and (iii) limiting downstream propagation reactions such as lipid peroxidation and protein/DNA oxidation [167]. In functional terms, this defense is designed to maintain ROS at “signaling-competent” levels for sperm maturation/capacitation while preventing transition into pathological oxidative stress.

#### 2.3.1. Enzymatic Antioxidants

Superoxide dismutase (SOD) constitutes a primary first-line defense by catalyzing the dismutation of superoxide (O_2_•^−^) into hydrogen peroxide (H_2_O_2_). Importantly, SOD activity is compartmentalized through isoforms: Cu/Zn-SOD (SOD1) is predominantly cytosolic, Mn-SOD (SOD2) localizes to mitochondria, and extracellular SOD (SOD3) contributes to antioxidant protection in extracellular/luminal spaces such as seminal plasma [168,169]. In human seminal plasma, SOD1 and SOD3 together account for most measurable SOD activity, consistent with the need to buffer diffusible oxidants surrounding sperm [170].

Because SOD activity increases H_2_O_2_, efficient H_2_O_2_-removal systems are essential. Catalase and glutathione peroxidases (GPx) detoxify H_2_O_2_ and lipid hydroperoxides, thereby restricting formation of highly reactive species and limiting membrane lipid peroxidation. Catalase activity is detectable in seminal plasma and has been reported to be reduced in some infertile phenotypes, supporting its relevance to sperm protection [171,172]. GPx enzymes are particularly important because they can reduce lipid hydroperoxides, which are central intermediates in chain-propagating membrane oxidation [165]. Among these, GPX4 is notable in male reproduction because it functions not only as an antioxidant enzyme but also as a structural component of the sperm midpiece (mitochondrial capsule), and disruption of mitochondrial GPX4 leads to male infertility with characteristic sperm midpiece defects [173].

Beyond SOD/catalase/GPx, sperm also express thiol-based peroxide-removal systems such as peroxiredoxins (PRDXs), which act as major sinks for peroxides and help maintain redox signaling during capacitation. In human sperm, PRDX activity is required to control ROS levels generated during capacitation and to prevent oxidative stress–associated loss of fertilizing competence [174].

Finally, enzymatic antioxidant capacity depends on reductant regeneration, particularly via the glutathione system. Reduction in peroxides by GPx consumes reduced glutathione (GSH), and restoration of GSH requires glutathione reductase and NADPH supply—linking sperm redox control to metabolic pathways such as the pentose phosphate pathway. This interdependence helps explain why antioxidant failure often presents as a network collapse rather than a single-enzyme defect [79,175].

#### 2.3.2. Non-Enzymatic Antioxidants

Non-enzymatic antioxidants provide complementary protection by directly scavenging radicals, interrupting chain reactions, and stabilizing membranes or chromatin. In seminal plasma, vitamin C (ascorbate) functions as a major aqueous-phase antioxidant, directly scavenging ROS and regenerating oxidized antioxidants, while vitamin E (α-tocopherol), as a lipid-soluble compound, acts as a chain-breaking antioxidant that protects sperm membranes from lipid peroxidation [176]. Vitamin E is considered a principal antioxidant within spermatozoa and plays an important role in maintaining membrane integrity and normal spermatogenesis, as vitamin E deficiency has been associated with abnormal germ cell development and impaired testicular function [177,178]. However, despite its clear biological relevance, clinical studies indicate that vitamin E supplementation alone has limited and inconsistent effects on improving overall semen quality [177]. Similarly, vitamin C contributes to membrane protection and redox buffering, and supplementation has been reported to modestly improve sperm motility and morphology in some studies, although its effects on sperm concentration and fertility outcomes remain controversial [176]. Vitamin B12 (cobalamin), while not a classical antioxidant, supports male reproductive function through its essential roles in DNA synthesis and cellular metabolism and has been associated with improved sperm concentration, motility, and reduced DNA damage [179]. Notably, vitamin B12 has also demonstrated protective effects against sperm cryodamage during assisted reproductive procedures, improving post-thaw viability and genomic integrity [180].

Coenzyme Q10 (CoQ10), present as ubiquinone and its reduced form ubiquinol, is a key component of the mitochondrial respiratory chain and an important lipid-soluble antioxidant in spermatozoa [181,182]. By facilitating electron transfer during oxidative phosphorylation, CoQ10 supports ATP production required for sperm motility, while its reduced form scavenges free radicals generated along the electron transport chain, thereby limiting mitochondrial ROS formation and protecting sperm membranes from lipid peroxidation [183]. Subfertile men frequently exhibit reduced seminal CoQ10 levels, which correlate with impaired sperm motility, morphology, and concentration [184,185]. Clinical studies, including randomized trials in oligoasthenoteratozoospermic (OAT) men, have shown that CoQ10 supplementation for at least three months can reduce oxidative stress, enhance antioxidant capacity, and improve selected semen parameters, although substantial variability in study design and outcomes precludes definitive conclusions regarding optimal dosage and universal efficacy [185,186].

Trace elements are essential “enablers” of redox defense. Selenium is required for multiple selenoproteins, particularly GPX4, which is essential not only for peroxide detoxification but also for the structural organization of the sperm mitochondrial sheath. Because the mitochondrial sheath supports flagellar energy production and midpiece integrity, selenium or GPX4 deficiency can impair mitochondrial sheath formation, resulting in structurally abnormal spermatozoa with reduced motility. Thus, selenium deficiency may impair sperm motility through both weakened antioxidant protection and defective GPX4-dependent midpiece/mitochondrial sheath formation [187]. Other selenoproteins, such as selenoprotein P (SePP), are highly enriched in the testes and seminal plasma and are required for normal spermatogenesis and sperm protection during epididymal storage and transit; seminal SePP levels positively correlate with sperm density and viability [188]. Clinical studies suggest that selenium supplementation at appropriate doses may improve semen parameters, including sperm concentration, motility, and morphology [189,190]. However, selenium exhibits a narrow therapeutic window, and excessive intake can disrupt physiological ROS signaling required for motility and acrosome reaction, leading to impaired sperm morphology and reduced motility [191,192].

In addition to classical antioxidant vitamins, CoQ10, selenium, and zinc, other redox-modulating agents have been explored in male infertility, including inositol-based approaches as adjunctive strategies for mitigating oxidative stress in male fertility [193]. N-acetylcysteine may support glutathione-dependent antioxidant defense, while L-carnitine and acetyl-L-carnitine may improve mitochondrial energy metabolism and sperm motility. Omega-3 polyunsaturated fatty acids contribute to sperm membrane fluidity, may reduce inflammatory oxidative stress, and have been reported to improve semen profile and enzymatic antioxidant capacity in infertile men with idiopathic oligoasthenoteratospermia [194], whereas lycopene, astaxanthin, melatonin, and selected polyphenols have been reported to exert antioxidant, anti-inflammatory, and mitochondrial-protective effects. Nevertheless, broader evidence on antioxidant therapy highlights important limitations, including context-dependent efficacy and the need to avoid non-specific empirical use [195]. Thus, antioxidant therapy should be applied cautiously and ideally guided by oxidative-stress phenotyping rather than used as non-specific empirical supplementation.

## 3. Molecular Mechanisms of ROS- and RNS-Induced Reproductive Damage

Excessive production of ROS and RNS disrupts cellular redox homeostasis and initiates a cascade of molecular events that compromise male reproductive function. Spermatozoa are particularly susceptible to oxidative and nitrosative injury due to their unique structural and functional features: (i) minimal cytoplasmic volume, which limits intracellular antioxidant buffering and repair capacity; (ii) high mitochondrial activity required for motility and related energy-demanding processes; and (iii) a plasma membrane enriched in polyunsaturated fatty acids (PUFAs) that is intrinsically prone to radical-driven chain reactions [7]. Importantly, the pathological impact of ROS/RNS extends beyond direct sperm damage to include impairment of genomic and epigenomic integrity, oxidation/nitration of proteins critical for motility and fertilization, mitochondrial dysfunction, and dysregulation of intracellular signaling pathways that coordinate spermatogenesis, epididymal maturation, capacitation, and fertilization.

### 3.1. Lipid Peroxidation and Sperm Membrane Integrity

Lipid peroxidation is among the earliest and most consequential outcomes of excessive ROS exposure in spermatozoa. The sperm plasma membrane contains abundant PUFAs that confer the membrane flexibility required for progressive motility, capacitation-associated remodeling, and sperm–oocyte fusion. However, the multiple double bonds within PUFAs make them highly vulnerable to radical attack—particularly by hydroxyl radicals and peroxyl radicals—initiating self-propagating oxidation chains [196,197]. Once initiated, lipid peroxidation generates lipid radicals and lipid hydroperoxides that fragment into reactive electrophilic aldehydes, including 4-hydroxynonenal (4-HNE), malondialdehyde (MDA), and acrolein [198,199]. These aldehydes are not merely by-products; they act as secondary toxic mediators that diffuse within the sperm cell and form covalent adducts with membrane and axonemal proteins, amplifying dysfunction even after the initial oxidant burst has passed.

Functionally, peroxidation-driven remodeling disrupts the physical and biochemical properties of the membrane, including membrane fluidity, lipid-raft organization, and receptor/ion-channel microdomains required for capacitation signaling. Oxidative injury can perturb Ca^2+^ homeostasis and downstream phosphorylation events essential for hyperactivation, thereby weakening flagellar beat patterns and reducing progressive motility [200]. Damage to the acrosomal and post-acrosomal membranes can also interfere with capacitation-dependent membrane fusion competence, contributing to defective acrosome reaction dynamics and impaired sperm–oocyte interaction. In addition, lipid peroxidation products can promote a feed-forward loop of oxidative stress by impairing mitochondrial membrane integrity and increasing electron leakage, further elevating ROS production and accelerating energy failure in the midpiece.

### 3.2. DNA Damage, Fragmentation, and Epigenetic Modifications

Sperm nuclear DNA is a central target of ROS- and RNS-mediated injury and represents a critical determinant of semen quality and male fertility. Although sperm chromatin is highly compacted through protamination—a specialization that confers substantial protection to the paternal genome—oxidative and nitrosative stress can nonetheless induce significant genomic damage [201]. Excessive ROS and RNS overwhelm antioxidant defenses and, together with dysregulated apoptotic processes, constitute the principal mechanisms underlying sperm DNA damage [202]. Oxidative stress can generate a broad spectrum of DNA lesions, including single- and double-strand breaks, DNA fragmentation, abasic sites, base modifications affecting purines, pyrimidines, and the deoxyribose backbone, as well as DNA crosslinking [203,204]. Among these lesions, oxidative base damage—most notably the formation of 8-hydroxy-2′-deoxyguanosine (8-OHdG)—serves as a widely accepted biomarker of redox-induced genomic injury in spermatozoa [205,206].

Reactive nitrogen species, particularly peroxynitrite formed by the rapid reaction of nitric oxide with superoxide, further exacerbate genomic instability through nitrative modifications and oxidative lesions that destabilize DNA structure and integrity [207]. The accumulation of such damage is especially consequential in spermatozoa, which possess minimal cytoplasmic repair machinery and therefore have a severely limited capacity to correct DNA lesions prior to fertilization. Consequently, oxidative DNA damage acquired during spermatogenesis or epididymal maturation is frequently transmitted to the oocyte. Clinically, elevated sperm DNA fragmentation—commonly quantified as a DNA fragmentation index—is consistently associated with reduced fertilization rates, impaired pre-implantation embryo development, decreased implantation success, and increased risks of miscarriage and adverse offspring outcomes [208,209]. At the molecular level, oxidative DNA lesions can interfere with gene transcription, promote replication errors, accelerate telomere attrition, and induce genomic instability, providing a mechanistic basis for the adverse reproductive and developmental consequences linked to sperm oxidative DNA damage.

Beyond direct DNA strand damage, redox imbalance can alter the epigenetic architecture of sperm. During spermiogenesis, chromatin undergoes extensive remodeling, including histone-to-protamine replacement and establishment of tightly packed DNA domains [210]. Oxidative stress can disrupt this remodeling, resulting in abnormal histone retention and altered distribution of histone-bound regulatory regions that are enriched near developmental genes [211]. Such perturbations may affect the delivery of paternal epigenetic information required for early embryogenesis. Oxidative stress has also been linked to altered DNA methylation patterns in sperm, potentially influencing gene regulation after fertilization [212,213,214]. Moreover, emerging evidence indicates that oxidative and metabolic stress can reshape sperm-borne regulatory cargo—such as small non-coding RNAs—providing additional routes through which paternal redox status may influence early embryonic programming [215]. Together, these observations highlight that ROS/RNS imbalance can compromise not only sperm viability and fertilizing capacity but also the molecular quality of the paternal contribution to embryo development, raising concern for effects that may extend beyond conception.

### 3.3. Protein Oxidation and Enzyme Inactivation

Proteins within spermatozoa and male reproductive tissues are highly sensitive to redox perturbations, and their function is critically dependent on the tight regulation of ROS and RNS levels [216]. Under physiological conditions, low and spatially controlled concentrations of ROS and RNS act as signaling molecules that modulate post-translational protein modifications essential for sperm capacitation, hyperactivation, and the acrosome reaction [217]. Superoxide anion, hydrogen peroxide, and nitric oxide participate in these processes by regulating phosphorylation cascades, particularly tyrosine phosphorylation of key sperm proteins. During capacitation, increased ROS availability is associated with enhanced tyrosine phosphorylation of specific proteins in the 70–105 kDa range, including the well-characterized 81 and 105 kDa phosphoproteins, which are essential for the acquisition of fertilizing capacity [218]. These phosphorylation events are coordinated through redox-sensitive signaling pathways involving protein kinase A (PKA), protein kinase C (PKC), mitogen-activated protein kinase (MAPK/MEK-like kinases), phosphatidylinositol 3-kinase (PI3K)/Akt signaling, intracellular Ca^2+^ fluxes, and cyclic AMP (cAMP)–dependent mechanisms [219,220,221,222].

However, when ROS and RNS production exceeds the buffering capacity of antioxidant defenses, signaling transitions into pathological protein oxidation and nitration. Excessive ROS induce oxidative modification of amino acid side chains, promote protein carbonylation, disrupt disulfide bond formation, and lead to protein misfolding or aggregation [223]. Concurrently, RNS—particularly peroxynitrite formed from the reaction of nitric oxide with superoxide—can nitrate tyrosine residues, altering protein conformation and impairing catalytic or structural function. Such irreversible oxidative and nitrosative modifications compromise proteins that are otherwise transiently regulated by reversible phosphorylation during physiological capacitation [224].

Oxidative damage to enzymes involved in energy metabolism, antioxidant defense, and signal transduction has profound functional consequences for spermatozoa. Oxidation of axonemal and peri-axonemal proteins disrupts coordinated flagellar beating, leading to reduced motility and impaired hyperactivation [225]. Inactivation of antioxidant enzymes, including superoxide dismutase, catalase, and glutathione-dependent systems, further exacerbates redox imbalance, creating a feed-forward cycle of oxidative injury [226]. Structural proteins within the sperm head and tail are also susceptible, contributing to abnormal morphology, compromised membrane dynamics, and defective sperm–oocyte interaction [227]. Specific examples illustrate how oxidative stress directly inactivates sperm proteins. Lipid peroxidation-derived electrophilic aldehydes, particularly 4-HNE, can target mitochondrial proteins and inhibit succinate dehydrogenase activity, thereby promoting mitochondrial electron leakage and further ROS generation [197]. In addition, exposure to H_2_O_2_ can induce sulfonation of sperm peroxiredoxins, including PRDX6, impairing their peroxide-removing capacity. High-molecular-mass complexes containing sulfonated PRDX6 have been detected in spermatozoa from infertile men, suggesting that irreversible oxidation of antioxidant enzymes contributes to persistent sperm redox dysfunction [228].

Importantly, oxidative stress can uncouple normal redox-regulated phosphorylation pathways from their physiological context. While moderate ROS levels promote capacitation-associated tyrosine phosphorylation and acrosome reaction through controlled kinase activation, excessive oxidative stress disrupts these signaling networks, suppresses phosphorylation fidelity, and interferes with acrosomal exocytosis [229]. This duality underscores the concept that ROS and RNS function as double-edged regulators of sperm protein function—indispensable for fertilization at physiological levels, yet profoundly detrimental when dysregulated—thereby linking protein oxidation and enzyme inactivation to impaired sperm function and male infertility.

### 3.4. Mitochondrial Dysfunction and Apoptosis

Mitochondria serve as both a principal source and a major target of ROSin spermatozoa, placing them at the center of redox-mediated sperm dysfunction [166]. Under physiological conditions, mitochondrial oxidative phosphorylation supplies ATP required for flagellar movement and fertilization-associated processes. However, excessive ROS and RNS generation damages mitochondrial membranes, respiratory chain proteins, and mitochondrial DNA, leading to impaired electron transport efficiency, reduced ATP production, and loss of mitochondrial membrane integrity. Experimental disruption of electron flow within the mitochondrial respiratory chain—particularly at complexes I and III—has been shown to trigger robust ROS generation in human spermatozoa [51]. Notably, ROS production at complex I on the matrix side of the inner mitochondrial membrane induces lipid peroxidation within the sperm midpiece and is closely associated with loss of motility, whereas ROS generation at complex III favors rapid hydrogen peroxide release into the extracellular space [51]. These findings highlight that the site and topology of mitochondrial ROS generation critically determine the extent of peroxidative damage and functional impairment.

Defective spermatozoa frequently exhibit spontaneous mitochondrial ROS production that is inversely correlated with motility, and quantitative analyses indicate that a substantial proportion of total cellular ROS variability in sperm can be attributed to mitochondrial sources [230]. Mitochondrial dysfunction, as assessed by impaired respiratory control ratios and altered bioenergetic profiles, is consistently associated with reduced sperm movement and fertilizing potential [231]. In parallel, oxidative and nitrosative stress induces post-translational modifications of mitochondrial and cytoskeletal proteins, including protein tyrosine nitration, which is predominantly localized to the sperm midpiece and head [232]. These nitro-oxidative modifications further compromise mitochondrial efficiency and axonemal function, reinforcing a vicious cycle of redox imbalance and energy failure. As mitochondrial performance declines, the resulting redox and bioenergetic instability extends beyond impaired ATP production to affect mitochondrial signaling functions that govern sperm survival and quality control.

In this context, mitochondrial oxidative stress emerges as a central regulator of apoptosis-like signaling in spermatozoa. In mature sperm, apoptosis-related processes—including loss of mitochondrial membrane potential, caspase activation, and phosphatidylserine externalization—are tightly coupled to mitochondrial status and are largely governed by mitochondrial integrity. Under physiological conditions, mitochondria generate regulated levels of mitochondrial ROS (mROS) that participate in capacitation-associated signaling and calcium-dependent pathways essential for fertilization [38]. However, excessive ROS and RNS overwhelm mitochondrial control mechanisms, leading to mitochondrial depolarization, outer membrane permeabilization, and release of pro-apoptotic factors such as cytochrome c, thereby activating caspase cascades [233]. Although spermatozoa possess a truncated apoptotic machinery and do not undergo classical apoptosis, oxidative damage to mitochondria triggers apoptosis-like degeneration that serves both as an active quality-control mechanism—limiting the fertilizing potential of DNA-damaged sperm—and as a passive consequence of irreversible mitochondrial injury [7,234,235,236]. These mitochondrial-driven apoptotic features, including phosphatidylserine exposure and DNA fragmentation, are frequently observed in infertile men and correlate strongly with impaired motility, abnormal morphology, and reduced fertilization capacity. Together, these findings position sperm mitochondria as integrative hubs linking redox signaling, calcium regulation, and apoptosis-like pathways, reinforcing mitochondrial dysfunction as a key mechanistic bridge between oxidative stress and male infertility.

### 3.5. Redox-Sensitive Signaling Pathways

ROS and RNS exert many of their biological effects through modulation of redox-sensitive intracellular signaling pathways, which play pivotal roles in maintaining normal spermatogenesis and sperm function while also mediating stress responses under pathological conditions. Among these, mitogen-activated protein kinase (MAPK) pathways are key redox-responsive regulators of cell proliferation, differentiation, survival, and apoptosis. In the male reproductive system, moderate ROS levels activate specific MAPK cascades—most notably p38 MAPK—to support physiological processes such as spermatogonial stem cell (SSC) proliferation and cellular adaptation to metabolic demand [237]. However, excessive or sustained oxidative stress leads to dysregulated MAPK activation, impairing germ cell homeostasis and somatic cell support functions [238]. In Leydig cells, aberrant p38 MAPK activation under oxidative conditions has been shown to suppress steroidogenic signaling by inhibiting steroidogenic acute regulatory protein (StAR) expression, thereby linking redox imbalance to reduced testosterone synthesis [239].

The nuclear factor kappa B (NF-κB) pathway represents another major redox-sensitive signaling axis within the male reproductive tract. Oxidative stress–induced NF-κB activation promotes transcription of pro-inflammatory cytokines, chemokines, and adhesion molecules, facilitating leukocyte recruitment and sustained inflammatory responses [97]. This inflammatory milieu further enhances ROS and RNS generation by activated immune cells, creating a self-amplifying cycle of oxidative stress that disrupts the testicular and seminal microenvironment and exacerbates sperm damage [240].

The phosphoinositide 3-kinase (PI3K)/AKT pathway is a critical pro-survival pathway in spermatozoa and is tightly regulated by cellular redox status [241]. In human sperm, components of the PI3K/AKT pathway and its negative regulator PTEN are compartmentalized into distinct cellular domains, enabling sustained production of phosphatidylinositol (3,4,5)-trisphosphate (PIP_3_) and promoting cell survival under physiological conditions [242,243]. Moderate ROS levels support PI3K/AKT signaling and mitochondrial integrity, whereas excessive oxidative stress disrupts this balance. Because mature spermatozoa are transcriptionally inactive and cannot undergo classical apoptosis, these changes are more accurately described as apoptosis-like degeneration. Inhibition of PI3K/AKT signaling induces rapid motility loss, mitochondrial ROS generation, caspase activation, phosphatidylserine externalization, and oxidative DNA damage [241]. This pathway is further regulated by PRDX6 calcium-independent phospholipase A_2_ activity, which helps maintain PI3K phosphorylation and sperm viability [244,245]. In addition, PI3K/AKT signaling can be supported downstream of lysophosphatidic acid receptor signaling in human spermatozoa, particularly through LPAR1, LPAR3, LPAR5, and LPAR6 [246]. Therefore, oxidative disruption of PI3K/AKT signaling links redox imbalance to loss of sperm viability and apoptosis-like features rather than classical apoptosis.

Collectively, these redox-sensitive signaling pathways illustrate how ROS and RNS function as context-dependent modulators of male reproductive biology. While tightly controlled redox signaling is essential for normal germ cell development, steroidogenesis, and sperm survival, excessive oxidative and nitrosative stress disrupts MAPK, NF-κB, and PI3K/AKT networks, shifting cellular responses from physiological adaptation toward inflammation, mitochondrial dysfunction, and apoptosis. The convergence of signaling dysregulation with lipid, protein, DNA, and mitochondrial damage provides a mechanistic framework linking oxidative stress to the diverse clinical manifestations of male infertility.

The principal cellular and environmental sources of ROS and RNS within the m ale reproductive system, together with their molecular mechanisms and clinical consequences, are summarized in Table 1. This table provides a concise overview of the major endogenous and exogenous sources of ROS/RNS, their dominant mechanisms of generation, key downstream molecular injuries, and representative reproductive outcomes. A more detailed version of this table, including expanded information, is provided in Supplementary Material S1.

## 4. Impact on Sperm Function and Fertility

Redox homeostasis is integral to normal sperm maturation and fertilization. Physiological levels of ROS and RNS act as signaling mediators that support capacitation-associated biochemical remodeling, acquisition of hyperactivated motility, and the acrosome reaction. When ROS/RNS production exceeds antioxidant capacity, however, oxidative and nitrosative stress shifts redox signaling from a regulated, reversible process to a damaging, self-amplifying state. This transition disrupts sperm function at multiple stages—from pre-fertilization signaling to gamete interaction—and is increasingly linked to both conventional semen abnormalities and impaired outcomes in assisted reproduction.

### 4.1. Disrupted Capacitation and Acrosome Reaction

Capacitation is a tightly controlled, multistep process involving plasma membrane cholesterol efflux, increased membrane fluidity, ion fluxes—particularly Ca^2+^ and HCO_3_^−^—elevation of cyclic AMP (cAMP) levels, and downstream protein tyrosine phosphorylation [247]. ROS and RNS are now recognized as integral components of this signaling network rather than mere by-products of metabolism. Physiological levels of ROS, especially hydrogen peroxide and nitric oxide–derived species, positively regulate capacitation by stimulating soluble adenylyl cyclase activity, enhancing cAMP production, activating protein kinase A (PKA) [222]. In parallel, ROS facilitate cholesterol oxidation and efflux from the sperm plasma membrane, a prerequisite for increased membrane fluidity and subsequent signaling competence. Redox modulation of protein tyrosine phosphatases further amplifies phosphorylation-dependent signaling, reinforcing capacitation-associated pathways [248].

Importantly, ROS-dependent capacitation is bicarbonate-sensitive, with HCO_3_^−^ acting upstream to coordinate ROS generation, cAMP signaling, and protein phosphorylation [249]. Experimental evidence across multiple mammalian species demonstrates that controlled oxidative conditions—whether generated endogenously by sperm mitochondria and oxidases or exogenously at low levels—enhance tyrosine phosphorylation, hyperactivation, and acrosomal responsiveness, effects that are reversibly suppressed by antioxidants such as catalase. Reactive nitrogen species, including nitric oxide and peroxynitrite, also participate in capacitation by inhibiting tyrosine phosphatase activity, activating SRC-family kinases, and stimulating oxysterol formation, thereby converging on shared redox-sensitive signaling nodes [249].

However, when ROS and RNS levels exceed physiological thresholds, this finely tuned signaling architecture becomes distorted. Excessive oxidative or nitrosative stress promotes indiscriminate lipid peroxidation, disrupts ion channel function, and induces aberrant or premature phosphorylation patterns that uncouple signaling fidelity from functional outcome. Rather than supporting capacitation, high ROS/RNS levels impair hyperactivation, compromise acrosomal exocytosis, and interfere with sperm–oocyte interaction [230]. Thus, capacitation exemplifies the dual nature of redox regulation in spermatozoa: a process that is fundamentally ROS-dependent, yet exquisitely vulnerable to oxidative imbalance, where deviation from optimal redox conditions rapidly translates into reduced fertilization potential.

Collectively, these findings highlight the central role of redox balance in regulating sperm capacitation. Physiological ROS and RNS levels function as signaling intermediates that integrate membrane remodeling, ion fluxes, and phosphorylation-dependent pathways required for the acquisition of fertilizing competence. However, because spermatozoa possess limited antioxidant defenses and a membrane highly enriched in polyunsaturated fatty acids, this redox-dependent signaling network is particularly vulnerable to oxidative perturbation. Even modest deviations from optimal ROS/RNS concentrations can disrupt the coordination of capacitation-associated pathways, leading to defective hyperactivation, impaired acrosomal responsiveness, and compromised sperm–oocyte interaction [217]. Thus, successful fertilization depends on the maintenance of a narrow redox window in which ROS/RNS support physiological signaling while excessive oxidative stress shifts the balance toward cellular dysfunction.

### 4.2. Defective Oocyte Binding and Fertilization Capacity

Successful fertilization requires the coordinated integration of progressive sperm motility, preservation of plasma membrane architecture, and the functional integrity of sperm surface proteins responsible for zona pellucida recognition, oocyte binding, and membrane fusion. Oxidative and nitrosative stress disrupts these prerequisites through several interconnected molecular mechanisms. Peroxidation of membrane lipids alters the physicochemical properties of the sperm plasma membrane, reducing membrane fluidity and destabilizing cholesterol- and sphingolipid-rich microdomains that serve as platforms for receptor clustering and signal transduction during sperm–oocyte interaction [251,252]. Such structural alterations interfere with the proper localization and activation of proteins involved in zona pellucida binding and gamete recognition.

In parallel, ROS- and RNS-mediated oxidative and nitrative modifications of proteins can impair the structure and activity of key surface receptors, ion channels, and signaling enzymes required for capacitation-dependent events, including the acrosome reaction and membrane fusion [253]. Oxidative damage to axonemal and cytoskeletal proteins further compromises flagellar function, leading to reduced progressive motility and defective hyperactivation, both of which are essential for successful penetration of the cumulus matrix and zona pellucida [254].

Beyond these structural and functional impairments, oxidative stress also affects the genetic integrity of spermatozoa. Although sperm carrying damaged DNA may still retain the ability to fertilize an oocyte, oxidative lesions and chromatin destabilization can compromise the quality of the paternal genome delivered to the zygote [255]. Such damage has been associated with impaired paternal genome activation, abnormal cleavage kinetics, reduced blastocyst formation, and increased risk of early embryonic arrest. Consequently, oxidative stress not only interferes with the fertilization process itself but may also negatively influence early embryonic development and reproductive outcomes. Together, these mechanisms illustrate how redox imbalance in spermatozoa can translate into reduced fertilization efficiency and diminished developmental competence of the resulting embryo.

### 4.3. Association with Clinical Infertility Phenotypes

Clinically, oxidative and nitrosative stress is frequently observed across multiple male infertility phenotypes and is increasingly recognized as a shared pathogenic mechanism rather than a condition confined to a specific semen abnormality. Elevated levels of reactive oxygen and nitrogen species have been reported in several common semen disorders, suggesting that redox imbalance contributes to diverse impairments in sperm production and function [217].

• Oligozoospermia—Increased oxidative stress has been linked to impaired spermatogenesis, potentially through oxidative damage to germ cells, induction of apoptosis within the seminiferous epithelium, and disruption of Sertoli cell metabolic and structural support. Redox imbalance within the testicular microenvironment may also impair Leydig cell steroidogenesis, indirectly affecting germ cell development [256].
• Asthenozoospermia—Reduced sperm motility is strongly associated with oxidative injury. ROS-mediated lipid peroxidation of the sperm plasma membrane alters membrane fluidity and ion channel activity, while mitochondrial dysfunction and oxidative modification of axonemal proteins compromise ATP production and flagellar movement [257]. These alterations impair both progressive motility and the hyperactivation required for effective fertilization.
• Teratozoospermia—Abnormal sperm morphology may arise from oxidative damage occurring during spermiogenesis and epididymal maturation. Excess ROS can disrupt chromatin condensation, interfere with protamine replacement, and impair cytoskeletal remodeling, resulting in structural defects of the sperm head, midpiece, or tail [258].

Importantly, oxidative stress is not restricted to men with abnormal semen parameters. Elevated oxidative stress markers and increased sperm DNA fragmentation have also been detected in individuals with apparently normal semen profiles, indicating that conventional semen analysis may not fully reflect underlying functional defects [172,259]. This observation helps explain cases of unexplained infertility in couples with normozoospermic male partners and highlights the potential clinical value of assessing oxidative stress biomarkers or sperm DNA integrity as complementary diagnostic tools in selected patients.

### 4.4. Effects on Assisted Reproductive Technology Outcomes

Oxidative and nitrosative stress not only impairs natural conception but also exerts measurable effects on outcomes in assisted reproductive technologies (ART). Elevated levels of seminal ROS and RNS, together with increased sperm DNA fragmentation and oxidative base lesions, have been consistently associated with reduced fertilization capacity and diminished embryo developmental competence. In conventional in vitro fertilization (IVF) cycles, oxidative damage to sperm membranes, mitochondria, and chromatin can compromise sperm–oocyte interaction and subsequent embryonic development [260]. Clinically, this may manifest as reduced fertilization rates, abnormal or delayed cleavage dynamics, impaired blastocyst formation, and lower embryo quality, particularly in cases where baseline sperm function is already suboptimal [261,262].

In intracytoplasmic sperm injection (ICSI), the mechanical bypass of sperm–oocyte interaction allows fertilization to occur even when sperm motility or membrane function is compromised. However, ICSI does not eliminate the biological consequences of oxidative sperm damage. Sperm carrying oxidative DNA lesions, chromatin instability, or epigenetic alterations may still fertilize the oocyte but subsequently impair embryonic genome activation and early developmental processes. As a result, elevated sperm DNA fragmentation and oxidative stress markers have been associated with reduced implantation rates, poorer embryo development, and increased risk of early pregnancy loss following ICSI [260,263].

Collectively, these observations position oxidative and nitrosative stress as important modifiers of reproductive success across the continuum of fertility care, from natural conception to ART. Understanding the molecular mechanisms through which redox imbalance affects sperm function and paternal genomic integrity is therefore critical for improving diagnostic evaluation in male infertility and for identifying patients who may benefit from targeted interventions, such as antioxidant therapy, lifestyle modification, or optimized sperm selection strategies during ART.

**Figure 1:**
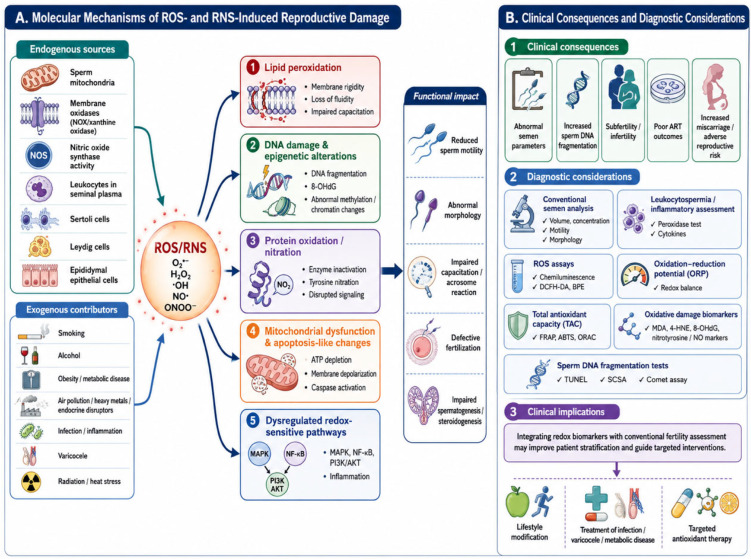
Molecular mechanisms and clinical consequences of ROS- and RNS-induced reproductive damage in the male reproductive system. Excessive ROS/RNS generation contributes to lipid, DNA, protein, mitochondrial, and signaling abnormalities, thereby impairing sperm function, spermatogenesis, and reproductive outcomes. Abbreviations: ROS, reactive oxygen species; RNS, reactive nitrogen species; NOX, NADPH oxidase; NOS, nitric oxide synthase; MDA, malondialdehyde; 4-HNE, 4-hydroxynonenal; 8-OH-dG, 8-hydroxy-2′-deoxyguanosine; ART, assisted reproductive technology; ORP, oxidation–reduction potential; TAC, total antioxidant capacity; MAPK, mitogen-activated protein kinase; NF-κB, nuclear factor kappa B; PI3K/AKT, phosphoinositide 3-kinase/protein kinase B; TUNEL, terminal deoxynucleotidyl transferase dUTP nick-end labeling; SCSA, sperm chromatin structure assay. (Created in BioRender. Wang, S. (2026) https://BioRender.com/u1026xj).

As summarized in Figure 1, redox homeostasis in spermatozoa represents a tightly regulated continuum, in which physiological ROS/RNS signaling supports key processes such as capacitation and acrosome reaction, whereas disruption by endogenous or exogenous stressors shifts this balance toward pathological oxidative and nitrosative damage.

## 5. Clinical Consequences and Diagnostic Considerations

Oxidative and nitrosative stress is increasingly recognized as a clinically relevant contributor to male infertility with implications for diagnosis, prognosis, and therapeutic decision-making. While conventional semen analysis remains the cornerstone of male fertility evaluation, it primarily assesses sperm concentration, motility, and morphology and may not fully capture underlying functional disturbances. Redox imbalance represents an additional biological dimension that can affect sperm competence even in the presence of apparently normal semen parameters. Consequently, the assessment of oxidative and nitrosative stress has emerged as a complementary diagnostic approach, particularly in cases of idiopathic infertility, recurrent pregnancy loss, or unexplained failure of ART.

### 5.1. Assessment of Oxidative and Nitrosative Stress in Semen

A variety of laboratory methods have been developed to evaluate oxidative and nitrosative stress in semen, reflecting the multifaceted nature of redox biology [264]. One commonly used approach involves the direct quantification of ROS using chemiluminescence-based assays. In these assays, luminol- or lucigenin-dependent reactions produce measurable photon emission proportional to ROS levels in semen samples or purified sperm suspensions. Chemiluminescence assays provide high analytical sensitivity and allow rapid quantification of global oxidative activity; however, the results may be influenced by leukocyte contamination, variations in sperm concentration, and differences in sample handling [265].

Complementary information can be obtained using flow cytometry–based techniques, which enable the detection of intracellular ROS production and oxidative damage at the single-cell level. Fluorescent probes such as dichlorodihydrofluorescein diacetate (DCFH-DA), MitoSOX Red, or BODIPY-based lipid peroxidation sensors allow assessment of cytoplasmic ROS, mitochondrial superoxide generation, and membrane oxidative injury within distinct sperm subpopulations [266,267]. These approaches provide valuable mechanistic insights but require specialized instrumentation and strict methodological standardization.

Additional diagnostic strategies focus on indirect markers of oxidative damage rather than direct ROS quantification. Measurements of lipid peroxidation products (e.g., malondialdehyde), total antioxidant capacity, or oxidative DNA damage biomarkers such as 8-hydroxy-2′-deoxyguanosine can reflect cumulative oxidative stress exposure [206]. Nitrosative stress may be evaluated through detection of protein nitrotyrosine residues, which represent stable footprints of peroxynitrite-mediated protein modification within reproductive tissues and seminal plasma [250].

Despite the availability of these assays, clinical implementation remains limited by methodological variability, lack of standardized reference ranges, and differences in reporting units across laboratories. Harmonization of testing protocols and the establishment of clinically validated cut-off values remain essential steps toward integrating oxidative stress testing into routine male infertility evaluation.

### 5.2. Correlation with Infertility Phenotypes

Elevated oxidative and nitrosative stress has been documented across multiple male infertility phenotypes and is often observed alongside abnormalities in semen parameters [268,269]. In men with oligozoospermia, increased ROS production may reflect disturbances in the testicular microenvironment, including impaired germ cell survival and altered Sertoli cell support during spermatogenesis. Asthenozoospermia shows a particularly strong association with oxidative stress, consistent with the susceptibility of sperm motility to mitochondrial dysfunction and structural injury affecting flagellar activity.

Importantly, oxidative stress is also frequently detected in men with idiopathic infertility, including those with normozoospermic semen profiles according to standard WHO criteria [270]. In such cases, redox imbalance may contribute to functional defects not captured by routine semen analysis, including reduced fertilization competence, compromised paternal genome integrity, or impaired early embryonic development. This observation underscores the potential diagnostic value of oxidative stress assessment as an adjunct tool in selected clinical scenarios, particularly in couples with unexplained infertility or repeated ART failure.

### 5.3. Predictive Value for Assisted Reproductive Technologies

Beyond its diagnostic relevance, the assessment of oxidative and nitrosative stress has gained attention for its potential prognostic value in ART. Increasing evidence indicates that elevated seminal ROS levels and biomarkers of oxidative damage—particularly sperm DNA fragmentation and oxidative base modifications—are associated with less favorable ART outcomes. In conventional IVF cycles, higher oxidative stress burdens have been correlated with reduced fertilization efficiency, altered embryo cleavage dynamics, and decreased blastocyst formation rates [260,271]. These associations appear particularly pronounced in cases where oxidative stress coexists with underlying semen abnormalities or elevated sperm DNA fragmentation indices, suggesting that redox imbalance may amplify existing sperm functional deficits that compromise gamete interaction and early embryogenesis.

In the context of ICSI, the mechanical introduction of a single spermatozoon into the oocyte bypasses several physiological barriers, including sperm motility and zona pellucida binding. Nevertheless, this procedure does not eliminate the biological consequences of oxidative damage to the paternal genome. Oxidative DNA lesions, chromatin instability, and epigenetic perturbations carried by the injected sperm may influence critical post-fertilization events, including paternal chromatin decondensation, zygotic genome activation, and early embryonic development [272]. Consequently, increased oxidative stress in sperm has been associated with impaired embryo quality, reduced implantation rates, and a higher risk of early pregnancy loss following ICSI in some clinical studies.

In addition to its prognostic implications, evaluation of oxidative and nitrosative stress may contribute to patient stratification and clinical decision-making in ART. Identification of elevated redox imbalance may prompt targeted interventions such as lifestyle modification, treatment of underlying inflammatory or infectious conditions, antioxidant supplementation, or the use of advanced sperm selection strategies designed to minimize the injection of oxidatively damaged spermatozoa [273]. Although the clinical utility of these approaches continues to be investigated, integration of redox biomarkers with conventional semen analysis and molecular assessments offers a promising strategy to improve risk prediction and individualized management of male infertility.

Overall, while methodological variability and the absence of universally accepted clinical thresholds remain challenges, the incorporation of oxidative and nitrosative stress assessment into the broader diagnostic framework of male infertility has the potential to enhance prognostic evaluation and optimize therapeutic strategies in ART.

## 6. Therapeutic Strategies and Interventions

Given the central role of oxidative and nitrosative stress in male reproductive dysfunction, therapeutic strategies aimed at restoring redox homeostasis have attracted increasing clinical interest. Current management approaches focus on reducing sources of oxidative injury, enhancing endogenous antioxidant defenses, and minimizing the downstream consequences of oxidative damage to spermatozoa. These interventions include lifestyle optimization, antioxidant supplementation, treatment of underlying medical conditions, and emerging pharmacological strategies targeting specific redox pathways. However, translating mechanistic insights from experimental models into standardized clinical therapies remains challenging. Considerable heterogeneity exists in patient populations, underlying etiologies of infertility, baseline oxidative stress burden, and methodological approaches used to assess redox status. As a result, the development of evidence-based and individualized treatment strategies continues to be an active area of research in reproductive medicine.

### 6.1. Lifestyle Modification and Reduction in Environmental Oxidative Stressors

Lifestyle optimization represents the most broadly applicable and low-risk intervention for reducing oxidative and nitrosative stress in men with infertility. Several modifiable behavioral and metabolic factors—including cigarette smoking, excessive alcohol intake, obesity, sedentary lifestyle, and poor dietary patterns—are known to increase systemic oxidative stress and impair reproductive function. Smoking cessation is particularly important, as tobacco smoke contains numerous pro-oxidant compounds capable of directly increasing ROS production and inducing DNA damage in spermatozoa [99]. Similarly, excessive alcohol consumption can disrupt mitochondrial metabolism and increase oxidative stress within reproductive tissues [274].

Nutritional optimization also plays an important role in maintaining redox balance. Diets enriched in fruits, vegetables, whole grains, and micronutrients provide natural antioxidant compounds, including vitamins, polyphenols, and trace elements that support endogenous antioxidant defense systems. Weight reduction and improved metabolic control may further alleviate oxidative stress by reducing chronic low-grade inflammation and improving endocrine function [275].

Regular physical activity is generally associated with improved metabolic health and enhanced antioxidant capacity; however, the intensity and duration of exercise are important considerations [276]. Moderate exercise may enhance endogenous antioxidant defenses, whereas excessive or high-intensity training has been associated with increased oxidative stress and potential impairment of spermatogenesis.

Reduction in environmental exposures is another important component of oxidative stress management. Limiting contact with air pollutants, heavy metals, pesticides, industrial chemicals, radiation sources, and endocrine-disrupting compounds may reduce exogenous ROS generation and mitigate cumulative oxidative injury to the male reproductive system [150,277]. In addition, appropriate management of medical conditions associated with increased oxidative stress—such as genital tract infections, metabolic disorders, and varicocele—may contribute to restoration of redox balance. Although lifestyle interventions alone may not fully reverse established sperm damage, they provide a foundational strategy that may improve overall reproductive health and enhance the effectiveness of adjunctive therapeutic approaches.

### 6.2. Antioxidant Therapy: Types, Dosage, and Clinical Evidence

Antioxidant supplementation remains the most extensively studied therapeutic strategy for oxidative stress–associated male infertility. A wide range of antioxidants have been investigated, including vitamins (e.g., vitamin C and vitamin E), mitochondrial cofactors such as coenzyme Q10, metabolic modulators such as L-carnitine and acetyl-L-carnitine, and trace elements including zinc and selenium [275]. These compounds act through complementary mechanisms, including direct scavenging of reactive species, regeneration of endogenous antioxidant molecules, stabilization of sperm plasma membranes, and support of mitochondrial bioenergetic function.

Clinical trials and meta-analyses have reported improvements in selected semen parameters following antioxidant supplementation, including increased sperm motility, improved concentration, and reductions in sperm DNA fragmentation and oxidative stress markers [278,279,280]. Some studies have also suggested potential benefits for pregnancy rates in couples undergoing natural conception or assisted reproduction. However, the magnitude and consistency of these effects remain variable across studies. Differences in antioxidant formulations, dosage regimens, treatment duration, and patient selection criteria contribute to significant heterogeneity in reported outcomes [281].

An additional consideration is the concept of redox balance, as excessive antioxidant intake may suppress physiological ROS signaling required for normal sperm function. This phenomenon, sometimes referred to as “reductive stress,” underscores the importance of avoiding indiscriminate antioxidant supplementation in unselected populations. Current evidence therefore does not support a universal antioxidant regimen for all infertile men. Instead, therapeutic benefit is most likely when antioxidant therapy is targeted toward patients with documented oxidative stress or specific clinical conditions associated with redox imbalance [273].

Future therapeutic strategies may increasingly focus on personalized approaches, integrating oxidative stress biomarkers with clinical and molecular data to guide treatment selection. Such strategies may improve the rational use of antioxidant therapies while minimizing unnecessary interventions and optimizing reproductive outcomes.

### 6.3. Emerging Molecular and Pharmacological Interventions

In addition to conventional antioxidant supplementation, emerging therapeutic strategies increasingly focus on targeted modulation of specific molecular pathways involved in redox regulation and sperm dysfunction. One promising area involves interventions aimed at improving mitochondrial function and bioenergetic stability, given the central role of mitochondria in sperm motility and redox homeostasis. Mitochondria-targeted antioxidants—such as coenzyme Q derivatives and other lipophilic redox-active compounds—have been developed to accumulate selectively within the mitochondrial matrix, where they can reduce mitochondrial ROS production and protect mitochondrial DNA, respiratory chain components, and membrane integrity [282]. Strategies aimed at enhancing mitochondrial quality control mechanisms, including mitochondrial biogenesis and mitophagy, are also being explored as potential approaches to improve sperm metabolic competence.

Another area of investigation involves anti-inflammatory therapies designed to reduce leukocyte-mediated oxidative and nitrosative stress within the male reproductive tract [283]. Because inflammatory activation of immune cells can generate substantial amounts of ROS and nitric oxide through NADPH oxidase and inducible nitric oxide synthase pathways, pharmacological approaches targeting inflammatory signaling may indirectly restore redox balance. These strategies may be particularly relevant in patients with genital tract infections, chronic prostatitis, or other inflammatory conditions associated with elevated seminal oxidative stress.

Advances in molecular pharmacology have also stimulated interest in modulating redox-sensitive signaling pathways that regulate cellular stress responses. Therapeutic approaches targeting pathways such as MAPK, NF-κB, or PI3K/AKT may offer greater specificity by restoring physiological signaling dynamics while limiting pathological oxidative responses [284,285]. Although such approaches remain largely experimental, they highlight the possibility of moving beyond generalized antioxidant therapy toward mechanism-based interventions tailored to the underlying molecular disturbances.

In parallel, innovations in nanotechnology and targeted drug delivery systems may improve the bioavailability and tissue specificity of redox-modulating compounds. Nanocarriers, liposomal formulations, and other delivery platforms have been investigated to enhance the stability and targeted distribution of antioxidant molecules within reproductive tissues. Despite these advances, most of these emerging strategies remain in preclinical or early translational stages, and rigorous evaluation of their safety, pharmacokinetics, and reproductive outcomes in humans is still required before clinical implementation.

### 6.4. Limitations and Gaps in Current Clinical Applications

Despite growing interest in redox-based therapeutic strategies, several challenges currently limit their widespread clinical application in male infertility management. One major limitation is the lack of standardized diagnostic criteria and validated biomarkers for identifying patients with clinically significant oxidative stress [286]. Although multiple assays are available to measure ROS production, oxidative damage markers, and antioxidant capacity, variability in methodology, sample preparation, and reporting standards complicates comparisons across studies and hinders the establishment of universally accepted clinical thresholds.

Interpretation of therapeutic outcomes is further complicated by heterogeneity in clinical trial design, including differences in patient selection, treatment duration, antioxidant formulations, and outcome measures. Many studies rely primarily on improvements in semen parameters rather than clinically meaningful endpoints such as pregnancy or live birth rates. Consequently, the true clinical impact of many redox-targeted therapies remains uncertain.

Another important consideration is the dual physiological role of ROS and RNS as both signaling molecules and mediators of cellular damage. Excessive suppression of reactive species may disrupt essential redox-dependent signaling processes involved in sperm capacitation and fertilization. This highlights the importance of therapeutic strategies aimed at restoring physiological redox balance rather than indiscriminately eliminating reactive species [273,287].

Finally, long-term safety data for many antioxidant and redox-modulating therapies remain limited [288]. Potential interactions between antioxidant supplementation and ART outcomes, as well as possible effects on epigenetic regulation and offspring health, are areas that require further investigation.

Addressing these limitations will require well-designed, large-scale clinical studies incorporating standardized oxidative stress assessment methods, clearly defined patient subgroups, and clinically relevant reproductive endpoints. Such efforts are essential for transitioning redox-based therapies from empirical use toward precision-guided and evidence-based management of male infertility.

## 7. Future Perspectives

Despite considerable progress in elucidating the roles of ROS and RNS in male reproductive biology, important challenges remain in translating mechanistic knowledge into clinically actionable strategies. Future advances will likely depend on the development of reliable biomarkers, improved integration of redox biology into fertility diagnostics, and the adoption of precision-based therapeutic approaches tailored to individual redox profiles and infertility etiologies. Continued interdisciplinary collaboration between basic scientists, clinicians, and bioengineers will be essential to bridge the gap between molecular discoveries and clinical application.

### 7.1. Emerging Biomarkers of Oxidative and Nitrosative Stress

A major priority for the field is the identification and validation of biomarkers that accurately reflect oxidative and nitrosative stress within the male reproductive system. Current diagnostic approaches often rely on global measures of ROS production or total antioxidant capacity, which provide limited mechanistic insight and may not capture the spatial and temporal complexity of redox regulation. Emerging biomarkers—including oxidative DNA lesions, lipid peroxidation products, protein nitration markers, and mitochondrial functional parameters—offer greater specificity and may better reflect biologically relevant oxidative injury in spermatozoa.

Future diagnostic strategies will likely benefit from multi-parameter biomarker panels that integrate multiple aspects of redox biology rather than relying on single indices [289]. In addition, advances in high-throughput technologies such as redox proteomics, epigenomics, metabolomics, and single-cell analysis may reveal novel molecular signatures linking oxidative stress to sperm functional competence and reproductive outcomes [290]. However, translation of these biomarkers into clinical practice will require rigorous validation, standardized laboratory methodologies, and the establishment of clinically meaningful reference ranges.

### 7.2. Integration of Redox Biology into Fertility Evaluation

Another key challenge lies in integrating molecular insights from redox biology into routine fertility assessment. Conventional semen analysis remains the cornerstone of male infertility evaluation but primarily reflects structural sperm characteristics rather than underlying molecular or functional defects [291]. Incorporation of oxidative and nitrosative stress assessment into diagnostic workflows may therefore provide complementary information regarding sperm functional competence and reproductive potential.

Such integration may be particularly valuable in clinical scenarios where standard semen parameters fail to explain reproductive failure, including unexplained infertility, recurrent pregnancy loss, and repeated failure of ART. Future diagnostic frameworks should emphasize the interpretation of redox-related findings within the broader context of patient-specific factors, including endocrine status, lifestyle and environmental exposures, and underlying medical conditions. The development of clinically validated diagnostic algorithms that incorporate oxidative stress biomarkers may facilitate more informed treatment selection and monitoring.

### 7.3. Targeted Therapeutic Strategies and Precision Reproductive Medicine

Advances in redox biology also open new opportunities for the development of targeted therapeutic interventions. Rather than relying solely on non-specific antioxidant supplementation, future strategies may focus on identifying and modulating specific sources of pathological ROS and RNS, such as mitochondrial dysfunction, inflammatory activation, or dysregulated oxidase activity within reproductive tissues [292]. Targeted mitochondrial therapies, selective modulators of redox-sensitive signaling pathways, and anti-inflammatory interventions represent promising areas for further investigation.

In parallel, the concept of precision reproductive medicine is gaining increasing attention. Personalized treatment strategies based on individual oxidative stress profiles, genetic susceptibility, environmental risk factors, and infertility phenotypes may improve therapeutic efficacy while minimizing unnecessary interventions. Achieving this goal will require longitudinal clinical studies, integration of molecular diagnostics with clinical data, and continued collaboration across disciplines including reproductive medicine, molecular biology, and bioinformatics.

Ultimately, advancing the clinical application of redox biology in male infertility will depend on the development of standardized diagnostic tools, well-designed clinical trials evaluating targeted interventions, and a deeper understanding of how oxidative and nitrosative stress interact with broader reproductive and systemic health.

## 8. Conclusions

ROS and RNS exert dual and context-dependent roles in male reproductive physiology. At controlled physiological levels, these reactive molecules function as critical signaling mediators involved in processes such as sperm maturation, capacitation, and fertilization. However, when ROS and RNS production exceeds the capacity of endogenous antioxidant defenses, oxidative and nitrosative stress can arise, leading to widespread cellular dysfunction. Excessive redox imbalance compromises sperm membrane integrity, genomic stability, protein function, mitochondrial bioenergetics, and redox-sensitive signaling pathways, collectively impairing sperm quality and reproductive competence. These molecular disturbances contribute to diverse clinical infertility phenotypes and can negatively influence reproductive outcomes in both natural conception and assisted reproductive technologies.

Recognition of the delicate balance between physiological redox signaling and pathological oxidative damage has important implications for the clinical management of male infertility. Incorporating redox-related assessments into fertility evaluation may complement conventional semen analysis by providing insights into functional sperm competence and underlying molecular defects. Such approaches may improve diagnostic precision, facilitate patient stratification, and guide more individualized treatment strategies. Nevertheless, despite promising advances in antioxidant and redox-targeted therapies, their routine clinical application remains constrained by variability in treatment protocols, heterogeneous patient responses, and the lack of standardized diagnostic and therapeutic frameworks.

Future progress in this field will depend on continued efforts to refine mechanistic understanding, establish reliable and clinically validated biomarkers, and develop targeted therapeutic interventions that restore physiological redox balance without disrupting essential signaling pathways. By integrating insights from molecular biology, clinical research, and reproductive medicine, the evolving study of redox regulation in male fertility holds significant potential to enhance diagnostic capabilities, optimize therapeutic strategies, and ultimately improve reproductive outcomes for affected individuals and couples.

## Figures and Tables

**Table 1:** Sources of Reactive Oxygen and Nitrogen Species (ROS: RNS) in the Male Reproductive System and Their Pathological Consequences.

Source/Compartment	Primary ROS/RNS	Dominant Mechanism of Generation	Key Downstream Molecular Injury	Representative Reproductive Outcomes	Key Refs in Review
Sperm mitochondria (midpiece)	O_2_•^−^, H_2_O_2_	Electron leakage from respiratory chain complexes I/III during oxidative phosphorylation; amplified by altered membrane potential and mitochondrial dysfunction	Lipid peroxidation, mtDNA damage, respiratory chain injury, feed-forward ROS amplification, apoptosis-like signaling	Reduced motility, impaired capacitation, lower fertilizing competence, higher DNA damage burden	[28,29,30,31,32,33,34,35,51,166,230,231,232,233,234,235,236]
Sperm membrane oxidases (NOX5 predominates; xanthine oxidase secondary)	O_2_•^−^, H_2_O_2_	Calcium-sensitive NADPH oxidase activity; xanthine oxidase contributes under purine dysregulation and cellular stress	Protein oxidation, membrane lipid oxidation, disruption of phosphorylation fidelity	Impaired capacitation, reduced sperm-oocyte interaction, fertilization failure	[36,37,38,39,40,41,42,217,218,219,220,221,222,247,248,249]
Nitric oxide synthase activity in sperm and reproductive tissues	NO•, ONOO^−^	NOS-derived NO• generation; excess NO• reacts with O_2_•^−^ to form peroxynitrite	Protein tyrosine nitration, mitochondrial dysfunction, lipid peroxidation, oxidative/nitrative DNA injury	Decreased motility and viability; defective fertilization-related signaling	[43,44,45,46,47,48,49,50,51,52,53,207,223,249,250]
Leukocytes in seminal plasma (neutrophils, macrophages)	O_2_•^−^, H_2_O_2_, NO•, ONOO^−^	Respiratory burst via NADPH oxidase and iNOS activation, coupled to cytokine-driven amplification	Diffuse lipid peroxidation, protein oxidation, nuclear and mitochondrial DNA damage, cytokine-amplified oxidative injury	Functional infertility despite normal routine semen parameters; increased DNA fragmentation and reduced sperm function	[54,55,56,57,58,59,60,61,62,63,64,65,66,67,68,69,70,71,72,73,74,148,149,150,151,152]
Sertoli cells	ROS (low-level), secondary RNS	Mitochondrial metabolism and redox-regulated signaling supporting germ cell differentiation and blood-testis barrier dynamics	Disrupted paracrine support, barrier dysfunction, impaired germ-cell microenvironment	Compromised spermatogenesis and lower sperm quality	[9,75,76,77,78,79,153,154,155,156]
Leydig cells	O_2_•^−^, H_2_O_2_	ROS generated during cytochrome P450-dependent steroidogenesis and mitochondrial microsomal metabolism	Steroidogenic enzyme dysfunction, mitochondrial injury, altered paracrine signaling	Reduced testosterone production, impaired spermatogenic support, endocrine contribution to infertility	[19,20,80,81,82,83,84,239]
Peritubular myoid cells	ROS (low-level)	Metabolic activity and regulation of seminiferous tubule contractility	Altered extracellular matrix composition and tubule dynamics	Indirect impairment of spermatogenic microenvironment	[85,86]
Epididymal epithelial cells/epididymal lumen	ROS/RNS at tightly regulated levels	Redox-dependent luminal control of membrane remodeling, disulfide bond formation, and chromatin stabilization	Membrane injury, chromatin packaging defects, oxidative DNA injury before ejaculation	Poor sperm maturation, reduced motility, compromised fertilization and ART outcomes	[87,88,89,90,91,92,93,94,95,96,97]
Cigarette smoking	Exogenous free radicals; secondary O_2_•^−^, NO•, lipid radicals	Direct oxidant exposure from tobacco smoke plus inflammation-mediated endogenous ROS/RNS generation	Lipid peroxidation, DNA fragmentation, mitochondrial dysfunction, leukocyte activation	Reduced motility, abnormal morphology, lower semen quality, higher genomic instability	[99,100,101,102,103,104,105,106]
Alcohol excess	ROS secondary to ethanol metabolism	Acetaldehyde production, altered NADH/NAD^+^ ratio, mitochondrial electron leakage, impaired antioxidant enzymes	Lipid peroxidation, membrane composition changes, Leydig/Sertoli dysfunction	Lower sperm concentration and motility, DNA damage, endocrine impairment	[104,105,106,107,108,109,110,111,112,113,114,115]
Obesity/poor diet/metabolic inflammation	ROS predominant; secondary RNS via inflammation	Adipokine-driven inflammation, mitochondrial dysfunction, free fatty acid excess, impaired antioxidant reserve	Lipid peroxidation, mitochondrial ROS excess, endocrine and spermatogenic dysregulation	Reduced semen quality, higher DNA damage susceptibility, impaired spermatogenesis	[116,117,118,119,120,121,122,123,124,149,150,151,152,153,154,155,156]
Air pollution/PAHs/traffic-related pollutants	ROS via redox cycling radicals	Metabolic activation to quinones/semiquinones, redox cycling, inflammatory activation of NADPH oxidases	Lipid peroxidation, oxidative DNA strand breaks, PAH-DNA adducts, altered chromatin packaging	Reduced sperm concentration and motility; increased DNA fragmentation	[125,126,127,128,129,130,131,132]
Heavy metals (lead, cadmium, mercury, arsenic)	ROS/RNS; hydroxyl radical-promoting chemistry	Mitochondrial electron transport disruption, trace element displacement, antioxidant enzyme inhibition, Fenton-like reactions	DNA strand breaks, lipid peroxidation, steroidogenic failure, epigenetic dysregulation	Impaired spermatogenesis, lower sperm quality, possible transgenerational risk	[133,134,135,136,137,138,139]
Radiation exposure	•OH, O_2_•^−^, H_2_O_2_	Radiolysis of intracellular water and secondary mitochondrial injury	Oxidative DNA damage, chromosomal instability, germ-cell injury	Reduced fertility potential and persistent sperm DNA damage	[140,141,142]
Endocrine-disrupting chemicals (phthalates, BPA, pesticides)	ROS/RNS	Mitochondrial dysfunction, altered Ca^2+^ homeostasis, pro-inflammatory activation, endocrine disruption	Sertoli/Leydig dysfunction, abnormal spermatogenesis, sperm DNA injury	Cumulative reproductive toxicity and declining male fertility	[143,144,145,146,147]
Male genital tract infection/inflammatory disease	O_2_•^−^, H_2_O_2_, NO•, ONOO^−^	Leukocyte recruitment, respiratory burst, iNOS induction, myeloperoxidase-dependent propagation	Protein nitration, lipid peroxidation, DNA oxidation, inflammatory amplification loop	Membrane injury, reduced sperm function, infertility not fully captured by routine semen analysis	[54,55,56,57,58,59,60,61,62,63,64,65,66,67,68,69,70,71,72,73,74,148,149,150,151,152]
Diabetes mellitus/hyperglycemia	ROS predominant; secondary RNS	Hyperglycemia-driven mitochondrial ROS excess, redox-sensitive pathway dysregulation, impaired antioxidant defenses	Membrane lipid peroxidation, DNA strand breaks/base oxidation, autophagy dysregulation, Sertoli/Leydig support failure	Reduced motility, increased DNA fragmentation, altered chromatin organization	[149,150,151,152,153,154,155,156]
Varicocele	ROS predominant; NO-related stress also reported	Hyperthermia, hypoxia/reperfusion-like stress, altered venous drainage, mitochondrial dysfunction	Lipid peroxidation, oxidative DNA damage, residual cytoplasm retention, reduced TAC	Abnormal morphology, lower sperm quality, infertility; partial biomarker reversal after varicocelectomy	[157,158,159,160,161,162,163,164]

## Data Availability

No new data were created or analyzed in this study. Data sharing is not applicable to this article.

## Supplementary Materials

The following supporting information can be downloaded at: https://www.mdpi.com/article/10.3390/antiox15070795/s1. Supplementary Table S1. Full version of the summarized Table 1 showing Sources of Reactive Oxygen and Nitrogen Species (ROS:RNS) in the Male Reproductive System and Their Pathological Consequences.
