# Supplementary material for: Reactive Oxygen and Nitrogen Species in Male Reproductive Health: From Molecular Mechanisms to Clinical Consequences
Source: Antioxidants (Basel). 2026 Jun 25;15(7):795. doi: 10.3390/antiox15070795 (PMC13403873; doi:10.3390/antiox15070795)
Supplement: Supplementary file 1 [file antioxidants-15-00795-s001.zip › antioxidants-4292878-supplementary.pdf]

Domain	Source / compartment	Dominant figure element inferred	Primary ROS/RNS species	Dominant mechanism of generation	Pathological amplifiers / clinical contexts	Key downstream molecular injury	Representative reproductive outcomes	Key refs in review
**Endogenous**	Sperm mitochondria (midpiece)	Central mechanistic node in sperm schematic	$O_2^{\bullet -}$, H_2O_2	Electron leakage from respiratory chain complexes I/III during oxidative phosphorylation; amplified by altered membrane potential and mitochondrial dysfunction	Immature or dysfunctional sperm, high ATP demand, aging, toxic exposure, heat stress, varicocele	Lipid peroxidation, mtDNA damage, respiratory chain injury, feed-forward ROS amplification, apoptosis-like signaling	Reduced motility, impaired capacitation, lower fertilizing competence, higher DNA damage burden	[28–35,51,166,230–236]
**Endogenous**	Sperm membrane oxidases (NOX5 predominates; xanthine oxidase secondary)	Signaling node adjacent to sperm plasma membrane	$O_2^{\bullet -}$, H_2O_2	Calcium-sensitive NADPH oxidase activity; xanthine oxidase contributes under purine dysregulation and cellular stress	Ca^{2+} dysregulation, inflammation, ischemia-reperfusion-like stress, defective sperm maturation	Protein oxidation, membrane lipid oxidation, disruption of phosphorylation fidelity	Impaired capacitation, reduced sperm-oocyte interaction, fertilization failure	[36–42,217–222,247–249]
**Endogenous**	Nitric oxide synthase activity in sperm and reproductive tissues	Nitrosative stress arrow converging on peroxynitrite	$NO^\bullet$, $ONOO^-$	NOS-derived $NO^\bullet$ generation; excess $NO^\bullet$ reacts with $O_2^{\bullet -}$ to form peroxynitrite	Inflammation, concurrent superoxide excess, dysregulated NOS activity	Protein tyrosine nitration, mitochondrial dysfunction, lipid peroxidation, oxidative/nitrative DNA injury	Decreased motility and viability; defective fertilization-related signaling	[43–53,207,223,249,267]

**Endogenous**	Leukocytes in seminal plasma (neutrophils, macrophages)	Inflammatory source node outside sperm, feeding ROS/RNS into seminal plasma	$O_2^{\bullet-}$, H_2O_2, NO•, $ONOO^-$	Respiratory burst via NADPH oxidase and iNOS activation, coupled to cytokine-driven amplification	Leukocytospermia, prostatitis, epididymitis, accessory gland infection, subtle inflammatory states	Diffuse lipid peroxidation, protein oxidation, nuclear and mitochondrial DNA damage, cytokine-amplified oxidative injury	Functional infertility despite normal routine semen parameters; increased DNA fragmentation and reduced sperm function	[54–74,148–152]
**Endogenous**	Sertoli cells	Testicular support-cell node	ROS (low-level), secondary RNS	Mitochondrial metabolism and redox-regulated signaling supporting germ cell differentiation and blood-testis barrier dynamics	Inflammation, toxic exposure, metabolic stress, oxidative overload	Disrupted paracrine support, barrier dysfunction, impaired germ-cell microenvironment	Compromised spermatogenesis and lower sperm quality	[9,75–79,153–156]
**Endogenous**	Leydig cells	Endocrine/steroidogenic node in testis panel	$O_2^{\bullet-}$, H_2O_2	ROS generated during cytochrome P450-dependent steroidogenesis and mitochondrial microsomal metabolism	Metabolic disease, inflammation, toxicants, chronic oxidative stress	Steroidogenic enzyme dysfunction, mitochondrial injury, altered paracrine signaling	Reduced testosterone production, impaired spermatogenic support, endocrine contribution to infertility	[19,20,80–84,239]
**Endogenous**	Peritubular myoid cells	Minor contextual node in testis microenvironment	ROS (low-level)	Metabolic activity and regulation of seminiferous tubule contractility	Chronic oxidative stress, extracellular matrix remodeling, tissue pathology	Altered extracellular matrix composition and tubule dynamics	Indirect impairment of spermatogenic microenvironment	[85,86]

**Endogenous**	Epididymal epithelial cells / epididymal lumen	Maturation compartment in tract schematic	ROS/RNS at tightly regulated levels	Redox-dependent luminal control of membrane remodeling, disulfide bond formation, and chromatin stabilization	Aging, epididymal inflammation, infection, environmental toxicants	Membrane injury, chromatin packaging defects, oxidative DNA injury before ejaculation	Poor sperm maturation, reduced motility, compromised fertilization and ART outcomes	[87–97]
**Exogenous**	Cigarette smoking	Clinical trigger box feeding both inflammatory and mitochondrial pathways	Exogenous free radicals; secondary $O_2^{\bullet-}$, NO•, lipid radicals	Direct oxidant exposure from tobacco smoke plus inflammation-mediated endogenous ROS/RNS generation	Current smoking, cumulative exposure, coexisting obesity or micronutrient deficiency	Lipid peroxidation, DNA fragmentation, mitochondrial dysfunction, leukocyte activation	Reduced motility, abnormal morphology, lower semen quality, higher genomic instability	[99–106]
**Exogenous**	Alcohol excess	Clinical trigger box linked to mitochondrial bioenergetics	ROS secondary to ethanol metabolism	Acetaldehyde production, altered $NADH/NAD^+$ ratio, mitochondrial electron leakage, impaired antioxidant enzymes	Chronic or excessive alcohol intake; synergy with smoking/obesity	Lipid peroxidation, membrane composition changes, Leydig/Sertoli dysfunction	Lower sperm concentration and motility, DNA damage, endocrine impairment	[104–115]
**Exogenous**	Obesity / poor diet / metabolic inflammation	Systemic metabolic trigger node	ROS predominant; secondary RNS via inflammation	Adipokine-driven inflammation, mitochondrial dysfunction, free fatty acid excess, impaired antioxidant reserve	Obesity, insulin resistance, antioxidant-poor diet, low testosterone/high estrogen milieu	Lipid peroxidation, mitochondrial ROS excess, endocrine and spermatogenic dysregulation	Reduced semen quality, higher DNA damage susceptibility, impaired spermatogenesis	[116–124,149–156]

**Exogenous**	Air pollution / PAHs / traffic-related pollutants	Environmental exposure box linked to systemic circulation	ROS via redox cycling radicals	Metabolic activation to quinones/semiquinones, redox cycling, inflammatory activation of NADPH oxidases	$PM_{2.5}$/PM_{10} exposure, PAHs, nitrogen oxides, urban traffic pollution	Lipid peroxidation, oxidative DNA strand breaks, PAH-DNA adducts, altered chromatin packaging	Reduced sperm concentration and motility; increased DNA fragmentation	[125–132]
**Exogenous**	Heavy metals (lead, cadmium, mercury, arsenic)	Toxic exposure box with direct gonadal toxicity	ROS/RNS; hydroxyl radical-promoting chemistry	Mitochondrial electron transport disruption, trace element displacement, antioxidant enzyme inhibition, Fenton-like reactions	Occupational/environmental exposure, chronic accumulation in testes/epididymis	DNA strand breaks, lipid peroxidation, steroidogenic failure, epigenetic dysregulation	Impaired spermatogenesis, lower sperm quality, possible transgenerational risk	[133–139]
**Exogenous**	Radiation exposure	DNA damage trigger box	•OH, $O_2^{\bullet-}$, H_2O_2	Radiolysis of intracellular water and secondary mitochondrial injury	Medical, occupational, radiotherapy-related, prolonged electromagnetic exposure	Oxidative DNA damage, chromosomal instability, germ-cell injury	Reduced fertility potential and persistent sperm DNA damage	[140–142]
**Exogenous**	Endocrine-disrupting chemicals (phthalates, BPA, pesticides)	Toxicant box with endocrine-redox cross-talk	ROS/RNS	Mitochondrial dysfunction, altered Ca^{2+} homeostasis, pro-inflammatory activation, endocrine disruption	Chronic low-level environmental exposure	Sertoli/Leydig dysfunction, abnormal spermatogenesis, sperm DNA injury	Cumulative reproductive toxicity and declining male fertility	[143–147]
**Exogenous**	Male genital tract infection /	Inflammatory clinical trigger converging on leukocyte node	$O_2^{\bullet-}$, H_2O_2, NO•, $ONOO^-$	Leukocyte recruitment, respiratory burst, iNOS induction,	Prostatitis, epididymitis, accessory gland inflammation,	Protein nitration, lipid peroxidation, DNA oxidation,	Membrane injury, reduced sperm function, infertility not	[54–74,148–152]

	inflammatory disease			myeloperoxidase-dependent propagation	intermittent/borderline leukocytospermia	inflammatory amplification loop	fully captured by routine semen analysis	
**Exogenous**	Diabetes mellitus / hyperglycemia	Systemic disease trigger feeding testis and sperm mitochondria	ROS predominant; secondary RNS	Hyperglycemia-driven mitochondrial ROS excess, redox-sensitive pathway dysregulation, impaired antioxidant defenses	Poor glycemic control, vascular oxidative stress, metabolic syndrome	Membrane lipid peroxidation, DNA strand breaks/base oxidation, autophagy dysregulation, Sertoli/Leydig support failure	Reduced motility, increased DNA fragmentation, altered chromatin organization	[149–156]
**Exogenous**	Varicocele	Clinical disease node connecting hemodynamic stress to mitochondria	ROS predominant; NO-related stress also reported	Hyperthermia, hypoxia/reperfusion-like stress, altered venous drainage, mitochondrial dysfunction	Clinical varicocele; oxidative burden worsened by concomitant infection/inflammation	Lipid peroxidation, oxidative DNA damage, residual cytoplasm retention, reduced TAC	Abnormal morphology, lower sperm quality, infertility; partial biomarker reversal after varicocelectomy	[157–164]
